# Livestock Multi‐Omics Integration: A Systematic Framework From Statistical Association to Causal Interpretation

**DOI:** 10.1002/advs.77094

**Published:** 2026-08-12

**Authors:** Jiying Wen, Zhongyu Wang, Jieping Huang, Fen Li, Ningbo Chen, Yun Ma

**Affiliations:** ^1^ Key Laboratory of Ruminant Molecular and Cellular Breeding of Ningxia Hui Autonomous Region College of Animal Science and Technology Ningxia University Yinchuan China; ^2^ College of Animal Science and Technology Guangxi University Nanning Guangxi China; ^3^ College of Animal Science and Technology Northwest A&F University Yangling China

**Keywords:** causal inference, livestock, machine learning, multimodal sequencing, multi‐omics integration

## Abstract

Livestock multi‐omics integration is key to unraveling complex trait regulation, yet systematic, livestock‐specific strategies remain scarce. This review traces the progression from single‐omics accumulation to multi‐dimensional integration, highlighting how large‐scale genomic, epigenomic, and transcriptomic projects lay the foundation for functional dissection. We identify core impediments: extreme species diversity, marked data heterogeneity, limited sample sizes, and a pervasive reduction of multi‐omics data to simplistic differential screens, resulting in low translational efficiency. We critically appraise four common pitfalls—overinterpreting correlation as causation, relegating proteomics to corroborating transcriptomics, incomplete microbiome–host integration lacking environmental context, and systematic neglect of metabolic fluxomics—and show how exposomics and fluxomics add necessary causal and dynamic dimensions. To address these, we propose a livestock‐adapted three‐tier analytical framework: (1) statistical association of cross‐omics covariation patterns; (2) machine learning‐driven feature mining and integrative modeling; and (3) causal interpretation encompassing Mendelian randomization, prior‐knowledge‐guided network inference, and physical causal evidence via fluxomics and metabolic control analysis. We further discuss how multimodal sequencing (single‐cell, spatial, temporal) and generative AI can fundamentally mitigate heterogeneity and strengthen causal evidence. Finally, we outline future priorities in database standardization, livestock‐specific benchmarking, and translational pipelines, charting a path from correlation‐centric reporting to mechanistic causality and precision breeding.

## Graphical abstract

A three‐tier livestock multi‐omics framework resolves four typical analytical pitfalls. Moving from statistical association through machine learning preprocessing to triple‐modal causal inference, it converts omics results into genomic selection and gene editing strategies to achieve One Health, underpinned by multi‐omics data, multimodal sequencing and applied AI.

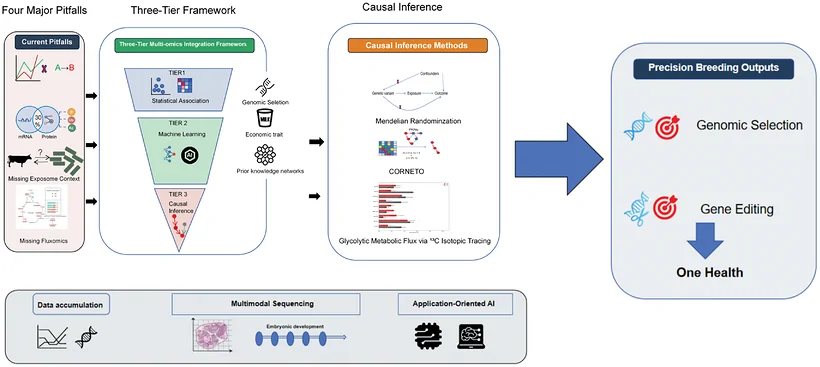

## 1 Introduction

Livestock underpin global food security and serve simultaneously as invaluable resources for human medical research and non‐model organism genetic studies [1]. The continuous advancement of multi‐omics technologies, organized around the central dogma as their core conceptual framework, has comprehensively expanded our understanding of the regulatory principles governing livestock genetics. Integrative multi‐omics analysis aims to obtain causal insights into biological phenotypes by combining data from genomics, epigenomics, transcriptomics, proteomics, metabolomics, fluxomics, and emerging technologies such as single‐cell and spatial omics [2]. The systematic integration of these multi‐dimensional data is crucial for dissecting the potential regulatory mechanisms underlying complex phenotypes in livestock [3]. However, comprehensive and systematic discussions of multi‐omics integration strategies and analytical methods tailored specifically for livestock remain notably scarce. The limited relevant literature either remains at the level of technical introductions to single‐omics approaches or discusses general difficulties commonly encountered in multi‐omics studies [4].

A prominent core challenge is the high diversity of livestock species, encompassing not only major production animals such as cattle, pigs, chickens, goats, and sheep, but also specialized economic animals including ducks, camels, and deer. Even within a single species, numerous breeds with significantly divergent genetic backgrounds exist [5]. Furthermore, the difficulty of obtaining target tissue samples from large animals, the high cost of sequencing, and the substantial variation in sequencing platforms and analytical methods adopted across different studies compound these challenges [4]. These issues are centrally manifested as pronounced data heterogeneity and imbalanced sample size distribution, and the frequent absence of platform‑standardized matrix data in public repositories, increasing the analytical complexity of livestock multi‐omics data integration [6]. With advances in artificial intelligence and the continuous development of sequencing technologies [7, 8], the development of novel multi‐omics integration methods adapted to livestock research scenarios represents a highly promising direction [9]. A systematic review and discussion of these methods are essential for defining the optimal integration pathways for livestock multi‐omics data.

In this review, we systematically trace the evolutionary trajectory of farm animal multi‐omics research from the accumulation of single‐omics data to multi‐dimensional systematic integration. We comprehensively dissect the prevalent cognitive misconceptions and core scientific challenges in multi‐omics integration, and extend the discussion to the core value and scenario applicability of exposomics and fluxomics in the field of livestock breeding research. On this basis, we provide an in‐depth discussion of the methodological spectrum of multi‐omics integration strategies and the core bioinformatics tools adapted for the functional dissection of livestock phenotypes. We operationalize a three‐tier integrative analysis framework tailored to livestock, comprising statistical association, machine learning‐driven feature selection, and causal inference. Concurrently, we explore the application prospects of artificial intelligence in livestock multi‐omics data analysis, and examine how single‐cell, spatial, and longitudinal multimodal sequencing technologies can provide robust data support for the elucidation of causal mechanisms. Finally, we present a systematic outlook on the future development pathways for livestock multi‐omics research, covering multiple core directions including the standardization of multi‐omics databases, the development of breeding scenario‐adapted tools and algorithms, and the industrial translation of multi‐omics achievements. Our aim is to provide a systematic theoretical reference for advancing the genetic dissection of complex traits and the innovative development of molecular breeding technologies in livestock, thereby contributing to the high‐quality and sustainable development of the global livestock industry.

## 2 The Omics‐Based Development and Data Accumulation Process in Farm Animals

Genomics is the core tool for genetic research in farm animals, and the continuously accumulating and expanding omics resources constitute the fundamental basis for the sustained development of the entire field [10]. Influenced by the Human Genome Project and its derivative initiatives [11], the rapid iteration of sequencing technologies and the development of international scientific collaboration have enabled major agricultural livestock species—cattle, pigs, chickens, and sheep—to progressively establish a complete technical system encompassing reference genome construction, population genetic variation analysis, and genome‐wide functional element annotation [12]. For instance, the 1000 Bull Genomes Project [13], animal pangenome research projects [14, 15], and continuous breakthroughs in sequencing technologies have particularly enabled the systematic discovery of previously intractable structural variants (SVs), including deletions, duplications, copy number variations, and chromosomal inversions, which have become critical sources of variation for dissecting breed differentiation and the genetic basis of complex traits in livestock [16]. Furthermore, the assembly of telomere‐to‐telomere (T2T) gapless complete genomes has allowed the comprehensive resolution of highly repetitive and complex genomic regions in species such as pigs, cattle, and sheep, supplementing numerous genes and sequence information absent from traditional reference genomes. Notably, the T2T gapless genome of the pig has fully resolved complex repeat‐rich regions, adding over a thousand novel genes missing from prior reference genomes [17]. The T2T complete assembly of the Y chromosomes of Bos taurus and sheep has, for the first time, precisely revealed their length differences, structural features, and patterns of gene divergence, clarifying that variation in ampliconic gene families is the core driver of Y chromosome size differentiation between species. This provides critical resources for understanding sex chromosome evolution in ruminants and for paternal genetic improvement in livestock [18]. Additionally, on this foundation, the FarmGTEx project has, through large‐scale multi‐tissue transcriptome sequencing, identified a vast number of tissue‐specific systematic cis‐expression quantitative trait loci (cis‐eQTLs). This effort currently covers core tissues of cattle [19], pigs [20], and chickens [21], with research progressively expanding to other livestock species including sheep, goats, horses, camels, rabbits, and ducks [22]. These key projects provide essential public resources for deciphering the genetic regulatory mechanisms underlying complex economic traits in livestock.

**FIGURE 1:**
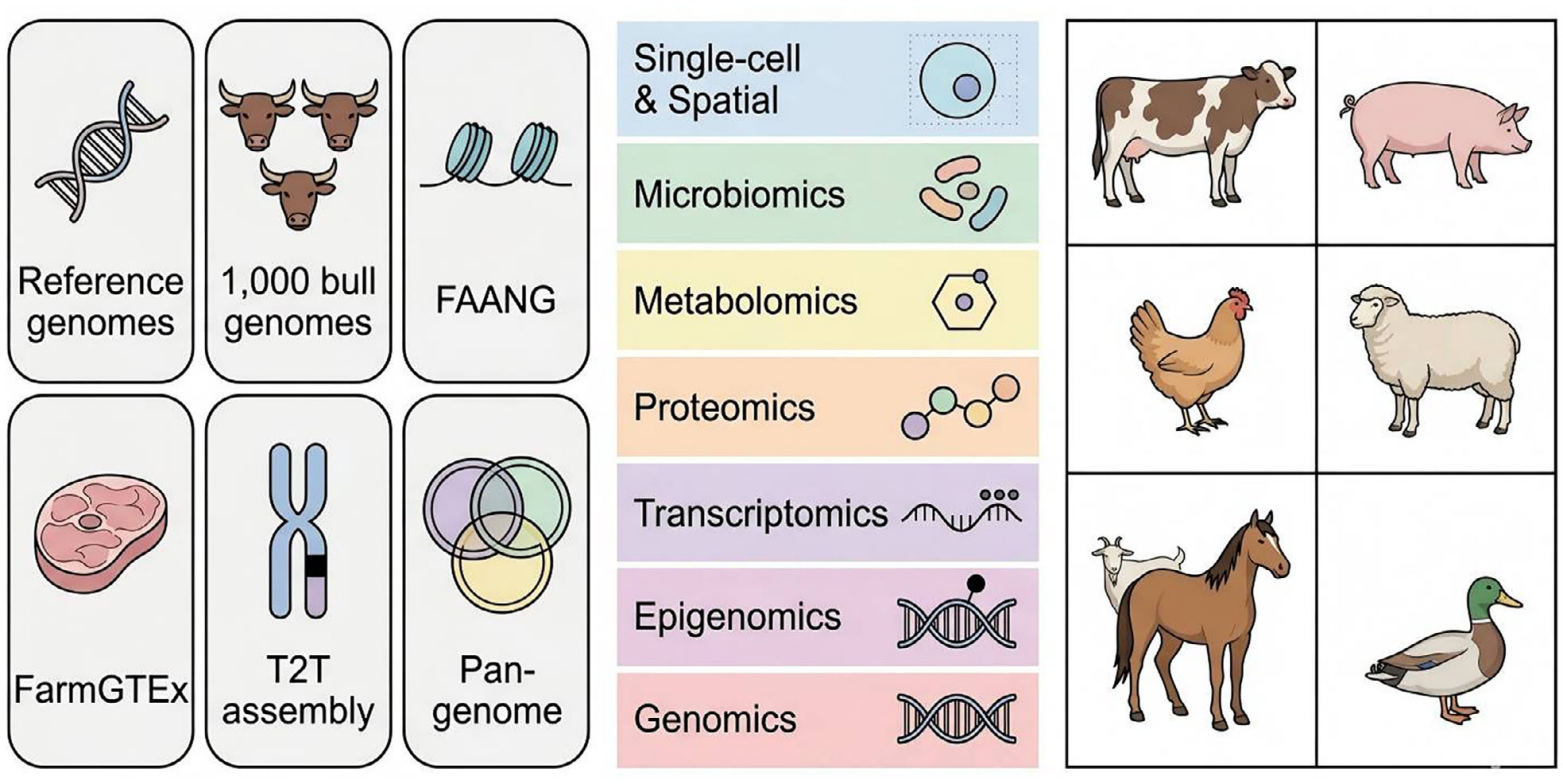
Milestones and data accumulation in livestock multi‐omics. Timeline of key projects and technologies (reference genomes, 1000 Bull Genomes, FAANG, FarmGTEx, T2T assemblies, pangenomes), stacked layers of omics dimensions (genome to spatial), and species coverage with data richness indicators.

At the level of functional annotation, international collaborative projects have propelled livestock genome research to accomplish the crucial transition from sequence interpretation to functional elucidation. The Functional Annotation of Animal Genomes (FAANG) consortium has integrated multi‐omics data, including ATAC‐seq, ChIP‐seq, and Hi‐C [23], to systematically map a comprehensive landscape of genome‐wide regulatory elements in major livestock species. In parallel, the omics characterization of indigenous livestock germplasm resources has also achieved significant strides. From the whole‐genome analysis of distinctive indigenous breeds such as the Butuo black sheep [24] and Guyuan cattle [25], to the construction of genetic resource repositories for various specialized livestock and poultry, the coverage and richness of farm animal genomic data resources have been comprehensively enhanced. With the continuous refinement and enrichment of genomic data resources, farm animal omics research is no longer confined to the single genome level but is progressively expanding toward multi‐omics integrative analysis, thus inaugurating the exploratory phase from single‐omics to multi‐omics. These steadily accumulating resources have also driven the development of specialized multi‐omics databases tailored to individual livestock species and cross‐species integration (Table 1), as well as core public repositories that host raw and processed omics data (Table 2). Together, they form the data infrastructure that supports the multi‐omics integration strategies discussed in later sections. The progressive accumulation of livestock multi‑omics data and the key technological milestones are summarized in Figure 1.

**TABLE 1:** A Systematic Inventory of Species‐Specific and Cross‐Species Standardized Multi‐Omics Databases for Livestock Multi‐Omics Integration Research.

Resource	Species	Dimensions	Highlights	Core Applications in Livestock	Website
FarmGTEx Portal	Cattle, pig, chicken, sheep, goat, buffalo and other major livestock	Multi‑tissue transcriptome, whole genome, methylation, eQTL/sQTL, splicing, single‑cell transcriptome	A flagship international consortium with standardized sample processing, sequencing, analysis pipelines and batch correction; currently the world's largest multi‑tissue, multi‑omics resource for livestock, eliminating data heterogeneities	Tissue‑specific eQTL mapping, candidate gene discovery for complex traits, genomic prediction optimization, cross‑breed regulatory conservation analysis	https://www.farmgtex.org/
FAANG Consortium Data Portal	Pig, cattle, chicken, sheep, goat, horse, etc.	Full‑spectrum epigenomics (ATAC‑seq, ChIP‑seq, Hi‑C), transcriptome, 3D genome, spatial omics, functional element annotation, single‑cell omics	The gold standard for functional genome annotation in livestock, defining unified classification, characterization and annotation of functional elements; directly fills the gap of lacking qualitative standards for functional elements; fully FAIR‑compliant	Annotation of regulatory elements in non‑coding regions, causal variant discovery for genotype‑phenotype associations, gene regulatory network construction, cross‑species functional conservation analysis	https://data.faang.org/
AnimalTFDB 4.0	All major livestock species	Transcriptome, transcription factor (TF) annotation, TF‑target regulatory network, epigenome binding sites	A comprehensive TF annotation database dedicated to livestock, with standardized TF classification, binding site prediction, and target gene regulatory validation; a core prior resource for constructing regulatory networks in livestock	Construction of trait‑associated gene regulatory networks, prior knowledge for causal inference, dissection of TF‑mediated regulatory mechanisms of traits	http://bioinfo.life.hust.edu.cn/AnimalTFDB4/
PigBiobank	Domestic pig (over 100 local and commercial breeds worldwide)	Genome, transcriptome, methylation, proteome, metabolome, gut microbiome, GWAS summary statistics, molQTL, TWAS/SMR data	Official database of the FarmGTEx‑PigGTEx project; a panoramic multi‑tissue, multi‑omics resource across the pig life cycle; standardized unified analysis pipeline integrating multi‑omics and GWAS data for meat quality, feed efficiency, disease resistance and other core traits	Multi‑omics integration for meat quality, feed efficiency, disease resistance; candidate gene screening; molecular marker development	http://pigbiobank.farmgtex.org/
CattleGTEx Portal	Cattle (beef and dairy)	Genome, multi‑tissue transcriptome, methylation, eQTL/sQTL, GWAS data, epigenome, TWAS data	First major output of the FarmGTEx project.standardized multi‑tissue regulatory variant analysis pipeline in cattle, covering candidate genes and regulatory loci for 43 economically important traits	Candidate gene discovery for milk production traits, mastitis resistance, growth and meat quality traits in beef cattle; genomic prediction optimization; TWAS analysis	https://cgtex.roslin.ed.ac.uk/
ChickenGTEx Portal	Chicken (broiler, layer, 123 breeds worldwide)	Genome, multi‑tissue transcriptome, methylation, epigenome, molQTL, single‑cell transcriptome, GWAS data	standardized multi‑tissue regulatory variant annotation pipeline in chicken, covering gene‑trait associations for 108 economically important traits	Multi‑omics integration for disease resistance, egg production, growth traits in poultry; molecular marker development; regulatory mechanism dissection	http://ngdc.cncb.ac.cn/chickengtex
iSheep Database	Sheep, goat	Genome, transcriptome, methylation, GWAS data, QTL annotation, phenotypic data, breed resource data	Official database of the International Sheep Genome Consortium (ISGC); standardized sheep/goat genome and multi‑omics analysis guidelines; covers core breeding trait data for major wool, meat and dairy sheep/goat breeds worldwide	Multi‑omics association analysis for wool traits, meat quality, milk yield, reproductive traits; candidate gene discovery; population genetics analysis	http://bigd.big.ac.cn/isheep/
Hungate1000 Collection	Ruminants (cattle, sheep, yak)	Rumen microbial metagenome, metatranscriptome, metabolome; host genome/transcriptome; microbial isolate culture data	Gold‑standard resource for rumen microbiome research in ruminants globally; standardized microbial genome assembly, annotation and functional analysis; integrates 410 complete rumen microbial genomes and massive metagenomic data	Host‑microbiome interaction mechanism dissection for feed efficiency, methane reduction, milk production traits; probiotic target screening	https://genome.jgi.doe.gov/portal/HungateCollection
Galbase	Chicken	Genome, transcriptome, epigenome, GWAS, QTL, genetic variation data	Currently the largest multi‑omics integration database for chicken; standardized variant annotation and multi‑omics joint analysis pipeline; covers transcriptome of 44 tissues, 379 epigenome datasets, over 15,000 QTLs	Genetic dissection of growth, egg production and disease resistance traits in chicken; multi‑omics integration analysis; functional marker development	http://animal.omics.pro/code/index.php/ChickenVar
CattleCA	Cattle	Single‑cell transcriptome, single‑nucleus transcriptome, spatial transcriptome, cell type annotation, cell‑cell communication network	the world's first single‑cell atlas covering 59 bovine tissues and 1.79 million cells; standardized single‑cell data analysis and cell annotation pipeline	Cellular heterogeneity dissection for tissue development; immune trait mechanism study; identification of cell types and target genes associated with economic traits	http://cattlecellatlas.farmgtex.org/

**TABLE 2:** Core Public Multi‐Omics Data Repositories for Livestock Research.

Resource	Species	Dimensions	Highlights	Website
NCBI GEO(Gene Expression Omnibus)	Cattle, pig, chicken, sheep, goat, etc.	Transcriptome (RNA‑seq), epigenome (ChIP‑seq, ATAC‑seq), single‑cell transcriptome, spatial transcriptome, sRNA, microarray	World‘s largest gene expression database, storing high‑throughput sequencing and microarray data. FAANG project data are accessible via GEO. Serves as a major archive for spatial transcriptomics, containing pig longissimus dorsi muscle (GSE161882), horse dorsal root ganglia, and other livestock spatial datasets	https://www.ncbi.nlm.nih.gov/geo/
NCBI SRA (Sequence Read Archive)	Cattle, pig, chicken, sheep, horse, goat, duck, etc.	Whole genome (WGS), transcriptome (RNA‑seq), single‑cell, epigenome, spatial transcriptome, metagenome	NCBI's core raw sequencing archive; stores raw data for FAANG and FarmGTEx projects. Central archive for spatial transcriptome raw sequence files; all livestock spatial transcriptome FASTQ files are deposited here.	https://www.ncbi.nlm.nih.gov/sra
NCBI BioProject / BioSample	All livestock species	Project‑level metadata and sample‑level metadata, linked to SRA/Assembly/other omics data	Provides standardized project registration and structured sample metadata; serves as the first‑level FAIR entry point. All high‑throughput projects (including spatial transcriptomics) must be registered here to obtain a project accession number.	https://www.ncbi.nlm.nih.gov/bioproject/ https://www.ncbi.nlm.nih.gov/biosample
ENA (European Nucleotide Archive)	Cattle, pig, chicken, sheep, goat, etc.	Whole genome, transcriptome, epigenome (ATAC‑seq, Hi‑C), spatial transcriptome, metagenome	Core nucleotide sequence repository at EMBL‑EBI; provides access to FAANG multi‑species multi‑omics data. Synchronized with SRA, also stores spatial transcriptome sequencing data with open access.	https://www.ebi.ac.uk/ena
PRIDE (Proteomics Identifications Database)	Cattle, pig, etc.	Proteome (LC‑MS/MS, TMT), modified proteomics (phosphorylation, glycosylation and other PTMs)	ProteomeXchange partner; publicly stores mass spectrometry proteomics data, including modified peptide identifications and spectral evidence. Representative datasets: PXD017837 (bovine mammary proteins), PXD016098 (calf muscle/plasma proteome).	https://www.ebi.ac.uk/pride/
MetaboLights	Cattle, pig, sheep, etc.	Metabolome (LC‑MS, GC‑MS, NMR), lipidome	EMBL‑EBI's cross‑species, cross‑technology metabolomics database, covering livestock metabolome and lipidome data.	https://www.ebi.ac.uk/metabolights/
ProteomeXchange	Cattle, pig, etc.	Proteome (mass spectrometry raw data)	Global proteomics data exchange consortium platform, integrating PRIDE, MassIVE and other nodes for unified mass spectrometry data access. Livestock‑related datasets include meat quality pH‑regulated proteome (lamb) and bovine rumen epithelium proteome.	https://proteomecentral.proteomexchange.org
GSA (Genome Sequence Archive)	Pig, chicken, etc.	Whole genome, transcriptome, epigenome	Core raw sequencing database at CNCB‑NGDC, archiving extensive domestic livestock omics data. Its sister database CROST provides dedicated spatial transcriptomics resources, integrating 182 high‑quality datasets, with some samples covering livestock tissues.	https://ngdc.cncb.ac.cn/gsa/
ADDAGMA (Domestic Animal Gut Microbiome Atlas)	Pig, cattle, horse, chicken	Gut microbiome (16S amplicon and metagenomic sequencing)	Integrates publicly available gut microbiome sequencing data from 356 published papers, covering 3,215 microbial taxa with 290,422 quantification events associated with 48 phenotypes. Provides customizable search and retrieval of sample metadata, experimental conditions, sequencing platforms, and microbial abundance.	http://addagma.omicsbio.info/

## 3 Initial Exploration and Challenges From Single‐Omics to Multi‐Omics

With the continuous development of sequencing technologies, the dimensions of accessible information have expanded beyond genomics to encompass the epigenome, transcriptome, proteome, metabolome, and other layers. Coupled with the application of emerging technologies such as single‐cell and spatial omics, the field of farm animal research has now accumulated a wealth of genetic data resources [26]. Thus, integrating multiple omics layers to dissect the mechanisms underlying animal genetic phenotypes is a major current focus in animal genetics.

Among these approaches, the integrated analysis combining transcriptomics and metabolomics is the most representative model. Specifically, in a study of Taihe black‐boned chickens, integrating the transcriptome and metabolome revealed dynamic changes in meat flavor compounds (ω‐3 polyunsaturated fatty acids, anserine, etc.) at different ages and their association with pathways such as focal adhesion and MAPK, thus revealing the age‐dependent molecular network underlying meat flavor [27]. Similarly, a study of Qiandongnan Xiaoxiang chickens used integrative analysis to identify sex‐specific metabolites and their associations with genes such as MAOA, clarifying that histidine and nicotinate metabolism are core sex‐related pathways and revealing the molecular basis of sex differences in meat quality [28]. In a nutritional regulation study of Tibetan sheep, integrative analysis revealed that combined dietary supplementation with resveratrol and HMB induced the upregulation of genes in the calcium signaling and NF‐κB pathways in the liver, which were co‐enriched with metabolites such as fumarate and leukotriene B4 in arginine synthesis and PPAR pathways; concurrently, serum IgM and antioxidant enzyme activities increased significantly. This elucidated the systemic mechanism by which nutritional intervention regulates immune and antioxidant functions through gene‐metabolite networks [29]. These studies fully demonstrate the core application value of integrated transcriptomic and metabolomic data analysis in dissecting complex traits in livestock. Currently, the majority of other multi‐omics analysis workflows either draw upon or directly adapt the transcriptome–metabolome joint analysis approach.

Multi‑omics integration can be classified into two major paradigms according to the flow of biological information: vertical integration and horizontal integration. Vertical integration follows the central dogma, combining genomics, transcriptomics, proteomics and translatomics to systematically dissect the linear information flow from DNA to protein. Beyond this main axis, metabolomics—as the ultimate phenotypic manifestation and material basis of gene function—is incorporated as a vertical extension, forming a complete chain from genotype to metabolic endpoint. Horizontal integration, on the other hand, expands upstream of the vertical information flow, focusing on functional elements such as epigenomics, 3D genome conformation, and non‑coding RNAs. This paradigm elucidates the molecular mechanisms of spatiotemporally specific gene expression at the regulatory level. In addition to the vertical integration centered on the central dogma, the multi‐omics integration of functional elements represents another major research direction for dissecting animal genetic mechanisms. Specifically, this involves the joint analysis of multi‐dimensional data on gene sequences, epigenetic modifications, and three‐dimensional genome conformation. For example, Cui et al. used CUT&Tag to identify the genomic targets of ZBTB16 in porcine immature Sertoli cells [30]. CUT&Tag is a next‐generation DNA–protein interaction technology primarily used to explore regions of histone modification, transcription factor binding status, and genome‐wide DNA–protein interactions. This exemplifies the integration of novel technologies with traditional genomics analysis. Furthermore, the regulation of non‐coding RNAs and RNA modifications (such as m6A methylation) have also emerged as novel research directions in livestock multi‐omics integration in recent years [31, 32].

Apart from the two integration perspectives mentioned above, the utilization of information brought about by improved sequencing resolution and additional dimensions constitutes another highly promising area in livestock multi‐omics research, exemplified by emerging technologies such as single‐cell sequencing and spatial omics. Single‐cell transcriptomics addresses the core limitation of bulk sequencing, which fails to distinguish heterogeneous cell populations within tissues. For instance, traditional bulk sequencing could only detect gene expression and chromatin states in porcine embryonic skeletal muscle tissue in a bulk manner, rendering it incapable of resolving cell‐type heterogeneity and differentiation dynamics during myogenesis. In contrast, integrating single‐cell transcriptomics with scATAC‐seq technology has systematically generated a single‐cell multi‐omics atlas of porcine embryonic somite and myotube development, constructed a differentiation trajectory of porcine skeletal myogenesis, and identified key transcription factors such as EGR1 and RHOB that regulate embryonic myogenic differentiation in pigs. This provides a high‐resolution core resource for dissecting the cellular dynamics and molecular regulatory mechanisms of skeletal muscle development in pigs [33]. Spatial transcriptomics technology, by virtue of its complete preservation of in situ spatial information within tissues, overcomes the technical limitation of traditional single‐cell sequencing, which loses information on the cellular spatial microenvironment. This provides novel research concepts and technical means for deciphering the phenotypic mechanisms of important traits such as reproduction and disease in livestock. For example, Jin, H. et al. used Stereo‐seq spatial transcriptomics technology to construct, for the first time, spatial transcriptomic atlases of bovine testicular tissue at two developmental stages: calf and adult bull. They identified 4 germ cell subtypes and 5 somatic cell subtypes, systematically dissected dynamic transcriptional changes during bovine spermatogenesis, and elucidated functional differences between cells and the communication networks among germ cells at different developmental stages. This work lays a core data foundation for understanding the mechanisms of spermatogenesis in large mammals and for the genetic improvement of male reproductive performance in livestock [34].

## 4 Low Efficiency in the Accumulation and Translation of Livestock Omics Data

Despite the comprehensive enrichment and accumulation of omics data related to livestock genetic resources, current omics research in the animal husbandry sector still faces the core bottleneck of low data integration efficiency and severely insufficient industrial translation rates. The rich array of multi‐omics data resources has far from realized its due scientific value and breeding application potential.

A pervasive negative tendency in the field is the reduction of high‐throughput omics data to a mere preliminary screening tool, resulting in an immense waste of data information. In the vast majority of published livestock omics studies, multi‐omics analysis is transformed into a preliminary screening method for candidate molecules based on classical statistical differential analysis. Taking combined transcriptomic and metabolomic analysis as an example, researchers often merely identify significantly differentially expressed genes or differentially abundant metabolites through simple intergroup comparison, and then declare the core data analysis pipeline complete. In doing so, they compress high‐throughput data—originally containing multi‐layered, rich biological information such as transcriptional regulatory dynamics, alternative splicing events, signaling pathway crosstalk, and metabolic process variation patterns—into a simplified list of differential molecules. This streamlined analysis model completely disregards the core value of omics data in dissecting the regulatory networks of complex economic traits such as meat production, milk production, reproduction, and disease resistance in livestock. The vast majority of the biological information contained in the data is directly discarded [35], fundamentally constraining the pathway from omics data to functional mechanistic elucidation and breeding application.

Furthermore, biased biological knowledge and over‑interpretation of statistical associations are only part of the explanation. A deeper reason is that current multi‑omics integration methodologies often consist of a simple transfer of single‑omics analysis modules. This largely stems from the tendency of many researchers to favor analysis workflows that are easy to run, rely on ready‑made code, and offer user‑friendly visualization, rather than statistically rigorous and biologically better‑adapted methods. At the same time, the number of sufficiently specialized peer reviewers is limited, which allows less rigorous analytical practices to become widely accepted as “default conventions” in the field. More fundamentally, not every method that appears reasonable can be directly transferred to the livestock context—even when the research goal seems identical to that in humans. Human toolchains rely on large‑scale reference atlases, tissue‑specific eQTL databases, and other infrastructure that is far more standardized than what is currently available for livestock. Therefore, one must be cautious about applying “pipeline‑style” large‑scale multi‑omics data integration methods and avoid forcefully transplanting ill‑adapted tools to livestock data.

Although genomics has already entered a mature development model characterized by large cohorts and pangenomics, non‐genomic omics data are constrained by sequencing costs and animal experiment expenses. In the majority of published studies, the total sample size for experimental and control groups is predominantly 6 or 12 [36, 37, 38]; only a minority of studies achieve larger sample cohorts [39], barely meeting the minimum requirements for statistical power. In some cases, solely to reduce sequencing costs, there is even an inconsistency in sample sizes across sequencing batches—for example, reducing the sample number for more expensive proteomics sequencing (only satisfying the minimum biological replicates) through sampling or pooling strategies [40, 41]. The direct consequence of small sample sizes is insufficient statistical power. On one hand, the inherent genetic background heterogeneity among individual livestock and minor fluctuations in the rearing environment can easily mask genuine biological treatment effects, causing core functional molecules strongly associated with the trait to be missed as false‐negative results [42]. On the other hand, when an adequate number of differential molecules cannot be identified under pre‐defined stringent statistical thresholds, a practice exists of arbitrarily relaxing statistical standards: downgrading from multiplicity‐adjusted FDR values to unadjusted raw P‐values, and possibly further relaxing core filtering thresholds such as the fold change (Log2FC) and significance level until the expected number of candidate molecules is obtained. Such non‐rigorous statistical strategies directly lead to a substantial increase in the proportion of false positives in the results [43]. Ultimately, apart from very few that can survive subsequent validation, the overwhelming majority of the candidate molecules screened in this manner lack biological reproducibility. Even for potential candidate molecules that are selected, a large amount of functional validation—at the cellular and molecular biology level, and at the live animal level—is still required before they can possess the initial potential to be translated into breeding molecular markers. The lengthy validation cycle combined with an extremely high validation failure rate ultimately results in the industry‐wide predicament of low translation efficiency for livestock breeding targets [44].

## 5 Common Issues in Systematic Multi‐Omics Integration

### 5.1 Overinterpretation of Correlation

First and foremost, correlation analysis provides a convenient and comprehensible pathway for integrative multi‐omics analysis in farm animals. However, a common misconception exists here: some studies equate correlation with causation, or treat correlation as synonymous with direct interaction. These are classic errors. For example, a study might find a significant correlation between gene A and metabolite B, and then report in the results: “Gene A interacts with metabolite B, leading to phenotypic changes.” Such a statement is easily falsifiable. The significant correlation between gene A and metabolite B might be a consequence rather than a cause, which is why the correlation is observed. Furthermore, genes and metabolites belong to different molecular dimensions; how gene A would affect metabolite B remains unknown. In a dataset comprising only 6–16 samples, the generalizability of such a correlation has also not been demonstrated. That is to say, based on the data alone, this constitutes a research hypothesis rather than a research conclusion.

### 5.2 Underutilization of Proteomics Function

As the ultimate executive molecules of genetic information, proteins constitute the core targets for the phenotypic dissection of livestock traits and can also provide functional molecular markers for precision breeding [45]. However, in current livestock multi‐omics research, proteomics is chronically relegated to a supporting role, used merely to corroborate findings from transcriptomics. Whether in studies on protein abundance changes related to meat tenderness [46], analysis of key enzyme activity regulation under stress conditions [47], or screening of trait‐associated biomarkers through inflammatory pathway activation during disease progression [48, 49], this analytical strategy, based on transcript–protein abundance correlation or concordance, is universally adopted.

Although intuitive and straightforward, this approach results in the substantial loss of core genetic information. Studies have demonstrated that only 30%–40% of protein variation can be explained by the corresponding transcript levels [50]. The biological processes spanning from mRNA to protein are far more than the linear translation process defined by the central dogma; a complex and dynamic layer of translational regulation exists between them. Translatomics is precisely the key technology for dissecting this core process. Centered on techniques such as ribosome profiling (Ribo‐seq), translatomics focuses on the entire dynamic processes of translation initiation, elongation, and termination. It can precisely resolve core translational events such as ribosome occupancy, translation elongation rate, cotranslational folding efficiency, upstream open reading frame (uORF) regulation, and ribosome stalling. Translatomics serves as the crucial intermediate bridge connecting the transcriptome and the proteome, and is the core entry point for explaining the discordance between transcript and protein abundance. However, current livestock multi‐omics research almost completely neglects regulatory information at the translatome level, providing only simplistic corroboration through mRNA–protein abundance correlation and entirely skipping the analysis of the translation process itself. Yet, dynamic changes in translation efficiency are precisely the core link determining the final abundance and function of proteins.

Furthermore, multiple layers of post‐transcriptional regulation also significantly influence protein expression and function, including alternative splicing of mRNA [51], targeted regulation by non‐coding RNAs [52], and post‐translational modifications such as phosphorylation, acetylation, and ubiquitination after translation is complete [53]. The redox state of the cellular microenvironment, metabolite concentrations, pH, and other factors can dynamically modulate protein function by affecting protein–ligand interactions and complex assembly [54]; even protein conformational changes can lead to gain or loss of function without any alteration in expression abundance [55]. This application model, which treats the proteome merely as corroboration for the transcriptome, not only completely loses genetic information from core regulatory layers such as translational regulation and post‐translational modifications, but also results in selected candidate molecules lacking direct functional support. This substantially increases the failure rate of subsequent functional validation, further exacerbating the industry‐wide predicament of low translation efficiency for livestock breeding targets. In essence, reducing proteomics to a passive validator of transcriptomics systematically discards the rich regulatory information encoded at the translational and post‑translational levels. This not only undermines the functional relevance of candidate molecules but also severs the causal chain between molecular change and phenotypic outcome.

### 5.3 The Perspective of Microbiome Multi‐Omics Integration Needs Refinement

Microbiomics is central to analyzing microbiota–host interactions in livestock. Its technical toolkit, including 16S rRNA gene sequencing, metagenomics, metatranscriptomics, metaproteomics, and metabolomics, provides comprehensive tools for investigating the composition, functional potential, and metabolic activity of microbial communities in the livestock gastrointestinal tract (rumen/gut). It has become a key focus in livestock nutrition, health management, and trait analysis [56]. However, the majority of current studies have merely established associations between the microbiome, the host endogenous regulatory system, and economic traits, lacking a systematic strategy grounded in deep biological insight. Microbiota–host interactions are often reduced to cross‐sectional statistical correlations, devoid of a clear causal regulatory chain. Most findings require rigorous interventional experiments and mechanistic validation before they can be applied in practice, rendering the biological connections delineated between microbiome and host multi‐omics data unstable. Exposomics offers a broader and more systematic framework. This concept, first proposed by Christopher Paul Wild in 2005 [57], encompasses the totality of non‐genetic environmental exposures an individual experiences from conception to death, along with the dynamic interactions between these exposures and the host biological system. From the exposomics perspective, analyzing microbiota–host functional interactions and integrating multi‐dimensional omics data by linking upstream environmental drivers, intermediate microbial mediation, and downstream host responses aligns better with the genuine biological process. Conclusions drawn using this approach possess stronger causal explanatory power and can more precisely dissect the regulatory mechanisms of complex economic traits in livestock. A noteworthy methodological exemplar is the study by Valdés‐Mas et al. published in Cell [58], wherein the authors systematically analyzed the tripartite interactions among dietary exposure, gut microbiota, and host intestinal physiology within an exposomic framework.

In livestock settings, the exposomics framework has demonstrated concrete operability. At the environmental exposure level, Seok et al. [59] performed a longitudinal multi‐omics analysis of finishing pigs integrating fecal metagenomics, whole‑blood transcriptomics, and immune cell deconvolution, tracking the dynamic changes of gut microbiota and host transcriptome under heat stress exposure. The study found that heat stress significantly reduced average daily gain and feed intake, decreased short‑chain fatty acid‑producing bacteria (e.g., Prevotella) while increasing Clostridium sensu stricto 1, and identified 516 differentially expressed genes enriched in pathways related to hematopoiesis, focal adhesion, cytoskeletal remodeling, and thyroid hormone signaling. Network analysis further identified cytoskeletal genes (ACTB, MYL9, ACTN4, COL4A4) as core regulatory nodes. This work systematically constructed a complete exposure‑response causal chain from environmental exposure (high temperature) to gut microbiota remodeling, to host gene expression changes, and ultimately to reduced production performance.

At the dietary exposure level, another study used alternative diet as an upstream perturbation variable in Nellore young bulls, integrating rumen and fecal microbiomes with host multi‑tissue transcriptomics [60]. Through gene co‑expression network and microbial co‑abundance network analysis, the study revealed that methane emissions under conventional versus alternative diets were associated with different microbial–host gene modules: under the conventional diet, methane emissions correlated with 42 host genes and Eubacterium; under the alternative diet, they correlated with 18 different host genes and Ruthenibacterium. This study not only demonstrated that dietary exposure reshapes the microbiome and host gene expression in a diet‑specific manner, but also established a complete response chain from dietary exposure to microbial metabolic changes, to host transcriptional responses, and finally to environmental phenotype (methane emissions). Guided by the exposomics framework, researchers can design controllable exposure experiments coupled with longitudinal sampling and systematic multi‐omics integration to identify interventional nodes along the complete chain from exposure to microbiota, from microbiota to host, and from host to phenotype, thereby providing decision‑making foundations for precision nutrition, environmental regulation, and stress‑resistant breeding.

### 5.4 Underutilization of Fluxomics in Livestock Multi‐Omics Integration

The problems discussed above focus largely on static omics (transcriptomics, proteomics, metabolomics)—misinterpretations of correlations, information loss between transcript and protein, and microbiome associations lacking environmental context—while neglecting the dynamic nature of cellular metabolism. Metabolic fluxomics, through stable isotope tracing combined with mathematical modeling, quantifies the true flow rates of carbon, nitrogen, and other elements in metabolic networks, serving as a critical bridge between static abundances and functional phenotypes. However, fluxomics is severely underutilized in livestock research, leaving our understanding of “how metabolism operates” at the level of static snapshots.

The uniqueness of fluxomics lies in the fundamental difference in the basis of its causal evidence from that of static omics. Static omics measure molecular concentrations; their data are essentially “observations,” and the causal inferences drawn from them depend on indirect reasoning pathways such as statistical correlations, prior knowledge networks, or genetic instrumental variables. Metabolic fluxomics differs fundamentally: its data come from the physical detection of stable isotope tracers—mass spectrometry (MS) or nuclear magnetic resonance (NMR) directly measures the physical transfer of atomic nuclei from one isotopic state to another [61]. When a ^1^ ^3^C‑labeled carbon atom moves from glucose to pyruvate to citrate, mass spectrometry detects not a “correlation” but the physical displacement of the atomic nucleus—the position and abundance of the tracer are constrained by physical‑chemical laws such as mass conservation, stoichiometric balance, and thermodynamic feasibility [62].

Thus, tracer detection is a physical‑level direct observation, not a statistical‑level indirect inference. The flux values output by ^1^ ^3^C‑based metabolic flux analysis (^1^ ^3^C‑MFA) are calculated through deterministic mathematical equations under the constraints of mass balance and isotopomer distribution. This means that the causal evidence provided by flux data is independent of statistical correlations—it does not answer “whether gene A is correlated with metabolite B” but directly answers “what is the net flow rate of carbon atoms from substrate X to product Y.” This characteristic gives fluxomics a unique position in the three‑tier framework of this review: it is a particularly valuable source of physical‑level causal evidence that is largely independent of statistical priors, which can be used to cross‑validate candidate metabolic nodes identified by statistical association and machine learning.

Bartman et al. systematically summarized three practical approaches for measuring fluxes in living mammals: arteriovenous differences (net organ flux), infusion of labeled tracers (whole‑body turnover flux), and measuring where tracer atoms end up in downstream metabolites [63]. Flux measurements can reveal regulatory phenomena that static omics cannot capture. For example, the turnover rate of a metabolite can change several‑fold while its concentration remains perfectly flat—a blind spot if one only measures abundance.

Wolfschmitt et al. used ^1^ ^3^C flux analysis in a pig model of shock to find that hyperoxia suppressed tricarboxylic acid (TCA) cycle flux in granulocytes, whereas conventional respiration and function tests detected no abnormality [64]. Beckett et al. combined fluxomics with proteomics in bovine kidney cells to show that pyruvate carboxylase levels dictate gluconeogenic capacity—and when saturated fatty acids inhibit this enzyme, cells are forced to fall back on amino acid anaplerosis [65].

From the perspective of causal inference, metabolic fluxomics, together with Metabolic Control Analysis (MCA), belongs to the family of causal inference frameworks [66]. Fell (2024) stated, the core feature of MCA is “to use mathematics to reason about the properties of biochemical pathways in a deductive manner” [67]. By quantifying control coefficients that measure the response of metabolic flux to small perturbations in enzyme activity, MCA directly answers counterfactual questions—such as “If we inhibit an enzyme by 10%, how much will the flux change?”—which embodies the operationalization of causal inference at the intervention and counterfactual levels.

In summary, the lack of fluxomics is a systematic deficiency in multi‐omics integration. The three methods proposed by Bartman et al. are fully transferable to livestock, but they are rarely adopted due to their technical demands. Flux information cannot be replaced by static omics and should be incorporated as an independent dimension alongside conventional omics layers. We recommend establishing standardized flux tracing protocols in livestock nutrition, immunity, stress, and breeding studies, and integrating flux data into multi‐omics models to advance the paradigm from “abundance association” to “metabolic causality.”

### 5.5 Current Difficulties and Challenges in Livestock Multi‐Omics Integration

The lack of omics‐scale perturbation databases for livestock limits the ability to conduct large‐scale benchmark testing and corresponding methodological development for various omics layers. While humans and mice possess rich perturbation resources—CRISPR screening databases, gene knockout phenotype databases, and drug perturbation multi‐omics databases [68]—such resources have long been severely deficient in livestock species. This situation is gradually changing: the development of a pig whole‑genome CRISPR/Cas9 knockout library (PigGeCKO, 85 674 sgRNAs targeting 17 743 protein‑coding genes) has provided a high‑throughput screening platform for pig functional genomics, and this library has been used to identify multiple host factors associated with Japanese encephalitis virus replication [69]. In cattle, a whole‑genome CRISPR knockout library (btCRISPRko.v1, targeting all protein‑coding genes in the bovine genome) has been constructed and successfully applied to screen host factors involved in bovine herpesvirus type 1 (BoHV‑1) replication, identifying several pro‑viral and antiviral candidate factors including GARP and EARP. CRISPR knockout library technology has also been extended to poultry, with whole‑genome sgRNA libraries providing new tools for identifying host factors for avian influenza virus infection [70, 71]. These advances collectively indicate that livestock perturbation resources are moving from “virtually non‑existent” toward “gradually accessible.” Nevertheless, validation in livestock multi‐omics studies still relies heavily on biochemical experimentation, and in most cases only one or two core pathways can be experimentally validated within a single study, with large‑scale systematic validation constrained by the lack of reliable data resources [44].

Furthermore, the qualitative characterization of functional elements remains a critical issue in multi‐omics integration. A vast amount of functional omics data carrying core regulatory information—including chromatin accessibility from ATAC/scATAC‑seq, spatial localization from spatial transcriptomics, post‑translational modifications from modification‑specific proteomics, transcription factor binding motifs, and three‑dimensional chromatin conformation from Hi‑C—is typically subjected only to coarse‑grained qualitative analysis due to the lack of standardized integration methods. Different qualitative classification strategies may lead to entirely disparate conclusions [72].

Recent livestock‑specific studies have generated rich functional genomic data but have also exposed a serious lack of standardized qualitative classification. At the 3D genomics level, integration of 71 Hi‑C datasets covering six pig breeds and five tissues constructed a pig genome‑wide 3D chromatin interaction map containing 65 843 topologically associating domains (TADs). Conserved TAD boundaries exhibit stronger insulation than breed‑specific boundaries, and genes within dynamic TADs play tissue‑specific roles [73]. High‑resolution 3D genome studies in pig liver and muscle further showed that tissue‑specific 3D structures have limited spatial overlap, and the formation of tissue‑specific chromatin loops correlates with chromatin accessibility, active histone modifications, and cooperative transcription factor enrichment [74]. Multi‑omics integration in pig muscle fiber type specification revealed that about 6% of the genome undergoes A/B compartment switching between breeds, with genes switching from inactive B to active A compartments enriched in pathways such as calcium ion transmembrane transport [75]. Subsequently, Tan et al. integrated transcriptomic, epigenomic, and 3D genomic data from slow‑ and fast‑twitch pig muscles and demonstrated that global remodeling of enhancer–promoter interactions drives transcriptional reprogramming associated with muscle contraction and glucose metabolism, with tissue‑specific super‑enhancers identified [76].

At the chromatin accessibility level, a comparative analysis of cattle, pig, and mouse across eight adult tissues identified 306 304 and 273 594 active regulatory elements in pig and cattle, respectively, with 71 478 pig and 47 454 cattle elements showing high tissue specificity and the most prevalent accessible motif being CTCF, suggesting its widespread involvement in 3D chromatin organization [77]. A whole‑organ ATAC‑seq peak catalogue in cattle compiled 976 813 cis‑regulatory elements and estimated that about one in three regulatory variants maps into an ATAC‑seq peak [78]. An ATAC‑seq atlas of 20 pig tissues identified 557 273 merged peaks and prioritized potential causal variants for polyunsaturated fatty acid content in muscle [79].

At the single‑cell level, the first single‑cell chromatin accessibility atlas of pig cerebral cortex and cerebellum identified nine major cell types and showed that pig regulatory elements are more conserved with humans than with mice [80]. Integration of single‑cell transcriptome and chromatin accessibility data from early gonadal development of goats, pigs, macaques, and humans revealed evolutionarily conserved and species‑specific cis‑regulatory elements and key transcription factors [81].

At the broader level of functional elements, super‑enhancers (SEs) and their 3D genomic organization have been systematically investigated in pig skeletal muscle and adipose tissue. Integrating SE annotation with ATAC‑seq, Hi‑C, and GWAS signals identified tissue‑specific SEs and their distal target genes, and functional variants regulating KLF6 (age at 100 kg), MXRA8 (lean meat rate), and TAF11 (loin muscle depth) were validated in pig populations [82].

**FIGURE 2:**
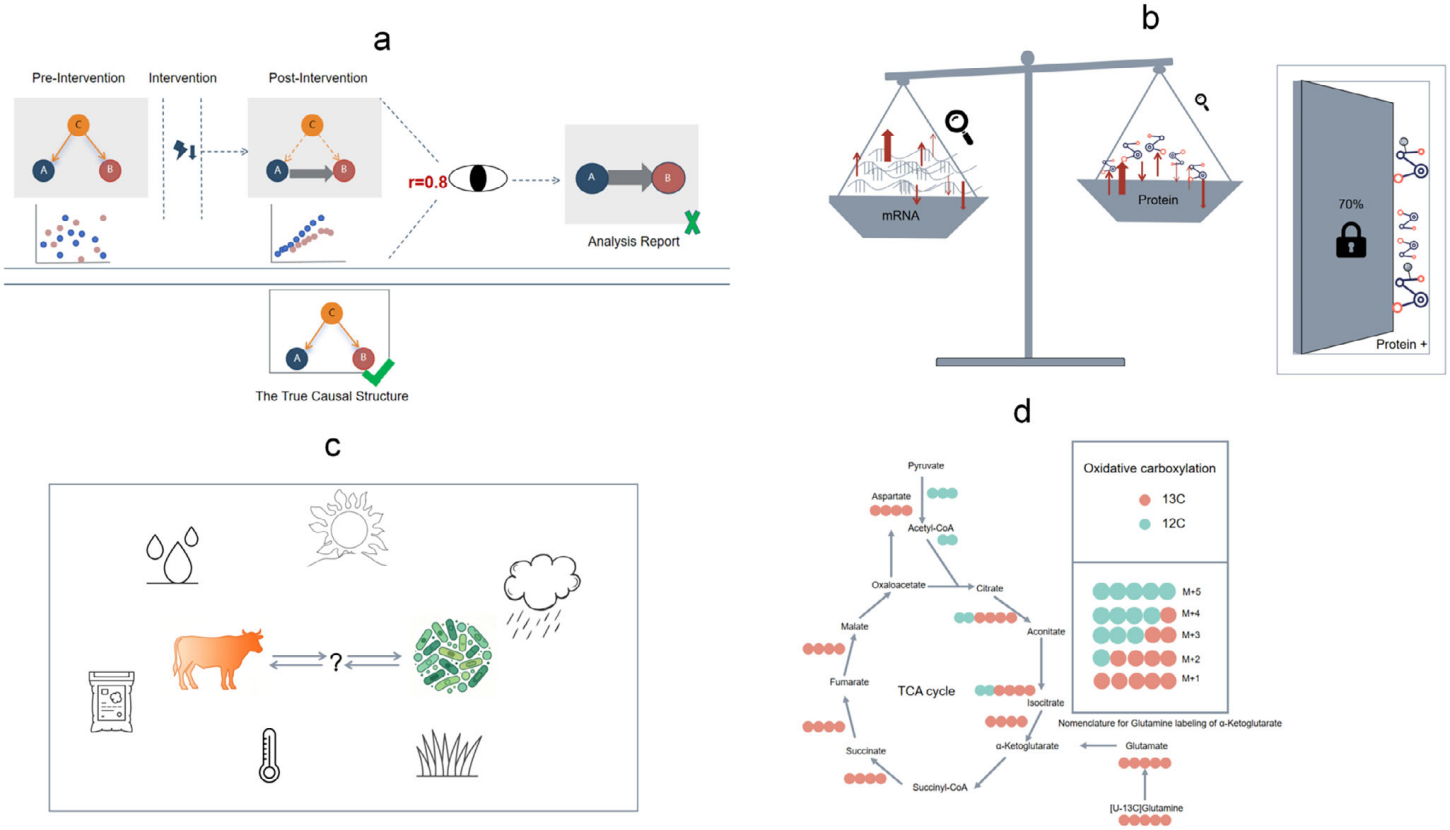
Major pitfalls and challenges in livestock multi‐omics integration. (a) Correlation is mistakenly interpreted as direct causality, ignoring latent confounding factors. (b) Proteomic data is reduced to transcriptomic validation, discarding post‐transcriptional regulatory layers that govern most protein variation. (c) Host‐microbiota multi‐omics fails to incorporate upstream environmental exposures from the exposome dimension. (d) Static metabolite abundance measurements cannot capture directional metabolic flow, while isotope‐based fluxomics delivers direct physical causal readouts of metabolic network dynamics.

Taken together, the qualitative analysis of functional elements in livestock faces a fundamental methodological gap. While Hi‑C, ATAC‑seq, and scATAC‑seq have generated massive, high‑dimensional functional genomic data across multiple tissues, breeds, and developmental stages, a unified, hierarchical qualitative classification standard for extracting standardized, comparable, and reproducible conclusions—such as whether a TAD boundary is conserved, whether a differentially accessible region is a genuine stage‑specific regulatory element, or whether a SE is a tissue‑specific candidate breeding target—is still lacking. Different research groups use different peak‑calling thresholds, conservation criteria, and TAD‑calling algorithm parameters, leading to divergent conclusions for the same biological question. The increasing richness of functional genomic data in livestock [72, 73] stands in stark contrast to the absence of a qualitative analysis framework. Therefore, prioritizing the development of novel integration strategies that incorporate structural, spatial, molecular interaction, and protein conformation information is essential to push the qualitative analysis of functional elements from “atlas reporting” toward “standardized classification.” Table 3 summarizes the common problems and challenges in current livestock multi‐omics data integration. The core challenges and common misconceptions discussed above are visually summarized in Figure 2.

**TABLE 3:** Core Challenges and Future Directions in Livestock Multi‐Omics Integration.

Challenge Type	Specific Manifestations	Potential Solutions	Expected Breakthroughs
Data Heterogeneity	Large differences across platforms, batches, and sample sources; inconsistent sequencing and analysis workflows	Establish standardized analysis workflows and sharing platforms [89], cross‐platform data calibration	Reduce data heterogeneity, unlock value of public data
Lack of qualitative standards for functional elements	Lack of qualitative standards for functional elements	Develop novel integration algorithms compatible with structural, spatial, and interaction information	Fill missing links between genotype and phenotype
Information omission	Only 30%‐40% of protein changes correlate with transcripts	Promote modified proteomics; develop precise biological insights into the translation process	Integrate proteomics into breeding selection models; improve the reliability of functional markers
Weak causal inference	Most studies emphasize correlation over causation	Develop causal inference algorithms adapted to livestock breeding scenarios	Move beyond association analysis toward mechanistic understanding; support precise intervention
Underutilization of fluxomics	Static omics dominate; metabolic dynamics ignored	Adopt stable isotope tracing (^1^ ^3^C MFA) and flux modeling in vivo/ex vivo	Move from “abundance correlation” to “metabolic causality”
Lack of perturbation resources	Scarce CRISPR, drug perturbation, or knock‑out databases for livestock	Build species‑specific Perturb‑seq resources; share multi‑omics perturbation data	Enable systematic functional validation; accelerate target discovery

## 6 The Methodological Spectrum of Multi‐Omics Analysis Strategies

**FIGURE 3:**
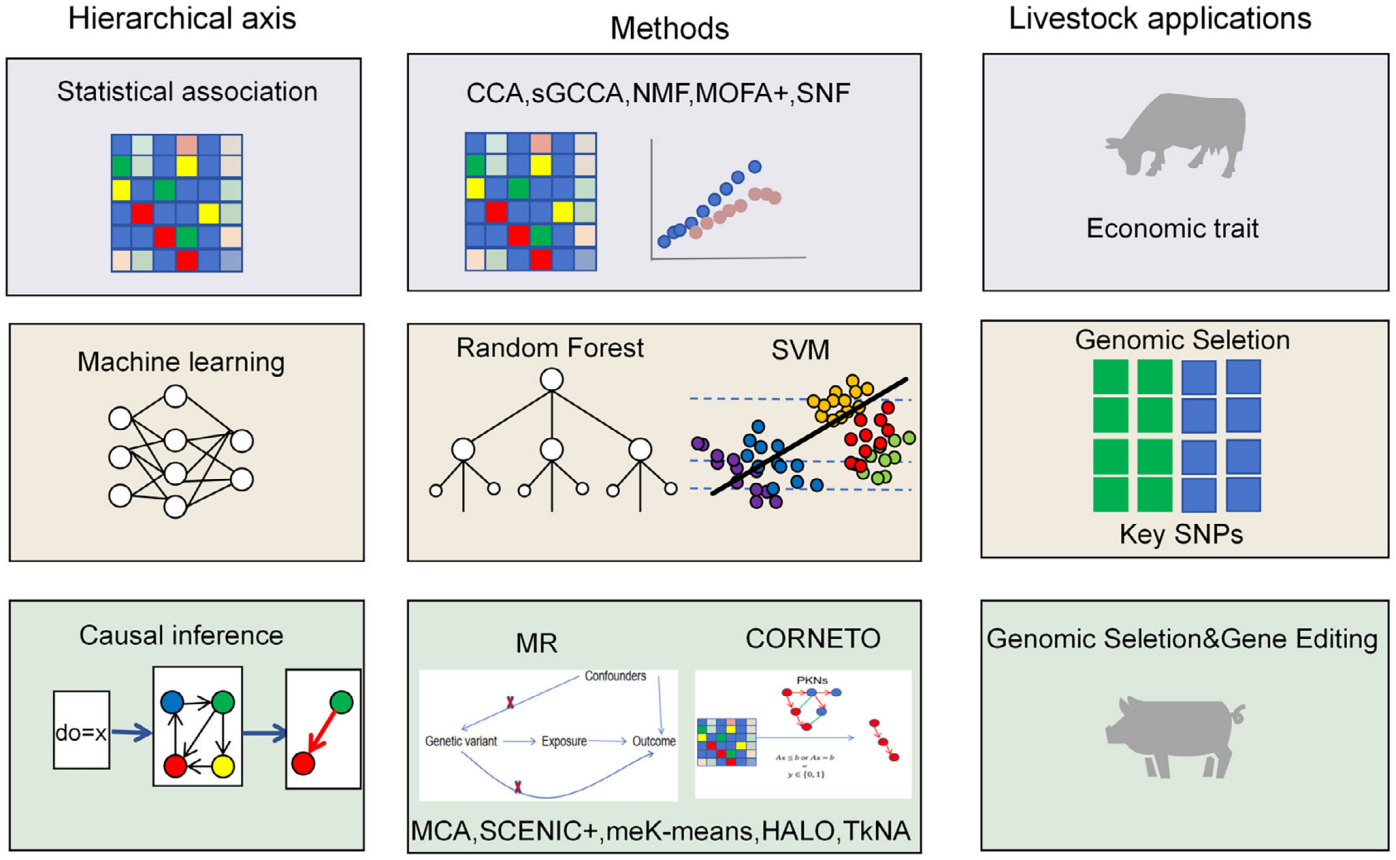
Three‐tier analytical framework for multi‐omics integration. The framework progresses sequentially from (1) statistical association to detect cross‐omics covariation associated with economic traits; (2) machine learning for denoising, feature prioritization and marker mining to boost genomic selection; and (3) formal causal inference to construct manipulable regulatory networks, whose results support both genomic selection and targeted gene editing for livestock molecular breeding.

Here we present a livestock‐adapted three‐tier analytical framework for multi‑omics integration, as illustrated in Figure 3. A substantial proportion of livestock multi‐omics studies commence with this category of methods.

### 6.1 Primary Integration: Statistical Association‐Based Identification of Cross‐Omics Covariation Patterns

As an entry‐level exploratory tier, primary integration aims to identify cross‐omics covariation patterns associated with the target trait, and to screen candidate molecules or pathways for further mechanistic validation. Currently, a substantial proportion of livestock multi‐omics studies commence with this category of methods. Its advantages include clear logic, intuitive understanding, low computational cost, and ease of standardization into a unified analytical pipeline. This tier also provides a convenient framework for dissecting the sources of heterogeneity underlying the same phenotype across different platform studies. For common economic traits with sufficient sample sizes, it enables the rapid screening of potential functional marker candidates.

Methodologically, integration at this tier generally relies on traditional statistical techniques such as correlation, covariance, matrix factorization, probabilistic models, kernel methods, or network fusion. Classical statistical methods such as Canonical Correlation Analysis (CCA) can explore linear associations between two sets of variables and have been widely used for the joint analysis of different genomic data types, such as DNA copy number variation and gene expression [83, 84, 85, 86, 87, 88]. For high‐dimensional omics data, extensions such as sparse CCA and sparse generalized CCA (sGCCA/rGCCA) significantly enhance model interpretability and stability through regularization constraints [86, 87]. Partial Least Squares (PLS) regression, another mainstream approach, achieves multi‐omics integration by maximizing the covariance between components [89, 90, 91].

Matrix factorization methods such as JIVE can decompose each omics matrix into joint and individual variation structures [92]; Non‐negative Matrix Factorization (NMF) and its extensions (jNMF, intNMF, LIGER) are more suitable for multi‐omics clustering and shared factor discovery [93, 94, 95, 96, 97, 98]. Probabilistic Bayesian methods, kernel methods, and network fusion approaches constitute another important category of technical pathways for multi‐omics integration within the realm of traditional statistics and unsupervised machine learning. Among these, probabilistic Bayesian methods can explicitly model uncertainty in the data and handle missing values automatically, and have been widely applied in multi‐omics studies [99], yielding favorable results in dissecting economic traits in livestock such as beef cattle [100, 101]. Kernel methods, through Multiple Kernel Learning (MKL), map different omics data into a unified kernel space, thereby effectively capturing nonlinear cross‐omics associations [102]; relevant studies have been successfully applied to genomic prediction in dairy and beef cattle [103, 104]. Network fusion methods construct integrative networks based on sample similarity, commonly used for population stratification and breed subtype identification, and have been validated in species such as pigs, beef cattle, and dairy sheep [105, 106, 107].

### 6.2 Second Tier: Machine Learning‐Driven Multi‐Omics Feature Mining and Integrative Modeling

As omics data scales increase and inter‐omics heterogeneity and missing values become more prominent, machine learning provides powerful tools for feature selection and integrative modeling. In this framework, machine learning is not positioned as an independent research objective, but as a methodological bridge that handles the complexity of livestock data before formal causal inference is attempted. In livestock multi‐omics, these tools serve three main purposes: (1) reducing dimensionality and visualizing complex data; (2) selecting informative features and discovering biomarkers; and (3) building predictive models while handling data heterogeneity and missing modalities.

For dimensionality reduction and visualization, principal component analysis (PCA), t‑SNE and UMAP are routinely used to simplify high‑dimensional microbiome or transcriptome data, making hidden structures and batch effects visible [108].

For feature selection and biomarker discovery, supervised learning methods such as LASSO regression and Random Forest effectively avoid overfitting and adapt to omics heterogeneity. They have been applied to identify trait‑associated markers in livestock [87, 88]. Unsupervised clustering methods (e.g., K‑means, hierarchical clustering, DBSCAN, consensus clustering) can stratify populations or discover functional microbial modules without requiring labels [106, 107, 109].

For predictive modeling and handling missing data, deep generative models—particularly Variational Autoencoders (VAEs) and their multimodal extensions—excel at learning joint latent representations from sparse, high‑dimensional multi‑omics data. They enable missing value imputation, batch effect correction, and cross‑omics translation [110, 111, 112]. VAE‑derived models such as MultiVI and scMM have been directly applied to microbiomics data [108, 113, 114, 115, 116, 117]. Regularization strategies including contrastive learning, adversarial training, and disentangled representation learning further improve generalizability and biological interpretability [118, 119, 120, 121, 122, 123].

Other deep learning architectures have also been explored. Generative Adversarial Networks (GANs), including omicsGAN, can generate cross‑modal data but suffer from training instability [124]. Mosaic integration methods (e.g., MIDAS, scVAEIT) address partially missing modalities through random masking or self‑supervised alignment [107]. More recently, Transformer‑based foundation models (e.g., scGPT, scFoundation) have shown strong transfer learning capabilities in single‑cell multi‑omics, offering a pre‑training and fine‑tuning paradigm that is particularly attractive for small‑sample livestock studies [124, 125].

Despite these advances, several challenges remain. Microbiomics integration still struggles with data heterogeneity, persistent batch effects, and limited model interpretability, calling for standardized workflows and reproducible validation frameworks [121, 122, 123].

Compared with primary statistical association methods, machine learning delivers clear advantages in nonlinear feature mining, heterogeneity modeling, and prediction accuracy. However, its application in livestock faces two core obstacles: selecting the right model for a specific economic trait, and overcoming the “black box” nature of complex models [126]. Livestock datasets are much smaller than human studies, genetic backgrounds are highly diverse across breeds and crossbreeds, and phenotyping systems are far from uniform. Therefore, practical applications must prioritize models with strong regularization, adopt transfer learning strategies, and strengthen interpretability analysis (e.g., SHAP values, latent space traversal) to ensure reliability and reproducibility.

### 6.3 Third Tier: Causal Interpretation of Multi‐Omics and Elucidation of Regulatory Mechanisms

Causal interpretation is the highest tier of multi‐omics integration and the ultimate goal of livestock research [127].While causal inference methods can also leverage machine learning tools, the distinction from the second tier lies in the analytical goal: causal inference aims to infer directionality, control confounding, and establish intervention‑ready causal links, whereas the second tier focuses on denoising, feature selection, and integrative modeling. By dissecting the core regulatory chains linking molecular changes to phenotypes, it aims to achieve precise intervention and genetic improvement of economic traits, moving beyond the common pitfall of “prioritizing correlation over causation.”

In livestock breeding, an operational causal claim rests on two complementary causal inference frameworks: the Rubin Causal Model (RCM) and Pearl's Causal Inference (PCI). RCM, centered on potential outcomes, defines the causal effect as the difference in possible outcomes for the same individual under two mutually exclusive treatments, formalizing causality as a missing data imputation problem [128, 129]. PCI, using structural causal models and directed acyclic graphs, answers “how the outcome would change if we forcibly alter a variable” through do‑calculus [130]. These two frameworks are complementary in livestock breeding: RCM is better suited for evaluating the causal effect of a single genetic variant or a specific breeding strategy, while PCI is more appropriate for constructing complex multigenic regulatory networks.

Based on the above frameworks, a meaningful causal claim must possess interventional interpretability—that is, the ability to predict how a hypothetical intervention (e.g., changing a genotype or selection scheme) would change the outcome. Three operational principles for evaluating the strength of a causal claim in livestock breeding are proposed: the counterfactual principle (the claim must imply a counterfactual: “if X were different, then Y would be different”), the intervention identification principle (the causal effect must be identifiable from observational data under reasonable assumptions or verifiable through active intervention experiments), and the breeding decision translatability principle (the causal finding must ultimately be translatable into actionable breeding decisions, answering “how much genetic gain would I obtain if I change this”).

Currently, causal inference methods applicable to multi‐omics data fall into three main categories. The first category is genetic‑variant‑based causal inference, represented by Mendelian randomization (MR). MR uses genetic variants as instrumental variables to infer causal effects of exposures on target phenotypes. Its validity depends on population‐specific GWAS and QTL summary statistics, which embed local linkage disequilibrium and allele frequency patterns. MR has been applied to dissect milk production and health traits in dairy cattle [131, 132]. However, its application in livestock faces four core species‑specific challenges: widespread population stratification in commercial breeds; weak instrumental variables due to small GWAS sample sizes for most non‑cattle species; horizontal pleiotropy in compact livestock genomes with extensive linkage disequilibrium; and a lack of tissue‑specific eQTL databases for most species beyond cattle and pigs. To address population stratification, principal component analysis and mixed linear models can be used, along with within‑family MR designs. In addition, we recommend including genomic inbreeding coefficients (FROH) as a covariate to control for inbreeding effects, and using multivariate MR to distinguish direct causal effects from confounding. For weak instrumental variables, summary‑data MR (GSMR) and Bayesian MR methods are recommended, together with cross‑breed GWAS meta‑analyses, strict LD clumping (r^2^ < 0.001) to retain independent variants, and bootstrapping for robust estimation. For horizontal pleiotropy, methods such as MR‑Egger, MR‑PRESSO, MR‑RAPS and colocalization analysis should be applied. For crossbreed studies, we suggest stratified MR (estimate within each breed then metaanalyze) rather than direct pooling. For missing eQTL databases, homology mapping from human/mouse data can serve as a temporary solution, and livestock‑specific eQTL integration communities should be established. Based on these challenges, we propose a five‑tier classification for MR applicability in livestock, explicitly stating that MR results should be treated as exploratory hypotheses when data are insufficient. The highest applicability applies to milk production and milk composition traits in dairy cattle, which have large‑sample GWAS and well‑established eQTL databases. The second tier applies to growth and reproduction traits in pigs, with moderately sized GWAS and partial eQTL data. The third tier applies to major economic traits in chickens and sheep, with small GWAS samples and limited eQTL information. The fourth tier concerns local or indigenous breeds, which are not currently recommended for MR but may become applicable as data accumulate. The lowest tier, where MR is not recommended at all, includes: (i) species with extremely poor genomic resources lacking reference genomes, population variation maps or LD reference panels, such as raccoon dogs (with reference genome but no GWAS and LD panels), meat pigeons (lacking breed‐specific GWAS and LD panels) and (ii) traits in mainstream livestock with controversial phenotyping, lacking large GWAS cohorts and tissue‑specific eQTL data, such as disease resistance (low‑incidence diseases), meat sensory traits (subjective assessment), behavioral traits, and lifetime traits. In such scenarios, the three core assumptions of MR cannot be satisfied, and MR is completely inapplicable. Any attempt must be considered exploratory and requires independent experimental validation.

The second category is prior‑knowledge‑driven causal network inference. These methods integrate prior biological knowledge—such as protein‑protein interactions and transcription factor–target gene relationships—to reverse‑engineer causal networks from downstream gene expression footprints. The priors used by these methods are experimentally validated physical molecular interactions, whose validity rests on biophysical laws conserved across organisms. CARNIVAL is a classic representative [133]; the 2025 updated CORNETO framework allows joint network inference across multiple samples [134]. SCENIC+ reconstructs the full “transcription factor → enhancer → target gene” causal chain by integrating chromatin accessibility, transcription factor binding, and gene expression [135]. A critical bottleneck is the lack of systematically curated, experimentally validated prior knowledge networks for livestock species. For example, even though SCENIC+ was published in Nature Methods in 2023, its application in livestock remains exploratory: Dufour et al. [136] applied the first‑generation SCENIC to porcine pre‑implantation embryo single‑cell data, but transcription factor motif associations were inferred through human‑pig homology rather than pig‑specific experimental networks [137].

For small‑sample, multi‑omics‑resource‑scarce local breeds, CORNETO is particularly recommended as a core tool. CORNETO's structured sparsity and cross‑sample joint inference design are especially suitable for “few samples, many dimensions” scenarios [134]. Its required prior knowledge network can be built using empirically validated cross‑species conserved regulatory relationships: Chen et al. systematically analyzed 137 epigenomic and transcriptomic datasets from six mammalian species (cattle, sheep, pig, mouse, human) and identified a core set of conserved regulatory elements in the liver, which showed significant concordance with GWAS signals across species [138, 139]; the RGD database further provides quantitative evidence that approximately 80% of human regulatory data can be successfully mapped to cattle, sheep, and goat genomes, with a recall of 82‑87% for promoter regions [140]. Based on these empirical data, the nearly impossible task of “causal inference on small‑sample local breed data” is transformed into a feasible, step‑by‑step engineering task: “joint cross‑sample inference under the constraints of a known conserved regulatory network.”

The third category differs from the first two. It does not rely on genetic variants or prior knowledge networks, but rather on the deterministic mathematical relationships governed by physical conservation laws. These methods directly quantify intervention effects through mass balance, stoichiometric constraints, and other physicochemical principles. A typical example is metabolic control analysis within fluxomics: using stable isotope tracers to measure metabolic fluxes, control coefficients directly quantify the extent to which a change in enzyme activity affects flux. In livestock breeding, this type of causal evidence can provide quantitative predictions for genetic interventions at key metabolic nodes—for example, predicting the flux increase of target products (such as milk fat or muscle amino acids) after up‑regulating a specific enzyme via gene editing. Together with the first two categories, it forms a complete causal evidence chain from statistical association to intervention effect to deterministic validation.

Emerging causal frameworks (HALO, meK‑means, TkNA) have recently expanded the toolbox into dimensions that classical MR and network inference cannot easily address. HALO [138] uses a causally regularized variational autoencoder to separate chromatin‑accessibility and gene‑expression changes into coupled and decoupled modes, applying Granger causality to identify distal regulatory interactions over time. meK‑means [141] takes a biophysical approach, modeling unspliced and spliced mRNA counts with chemical master equations to define cell states through physical causality. TkNA [142] integrates multi‑omics data from multiple independent cohorts, filtering spurious associations via correlation inequalities and identifying master regulator nodes through network topology metrics. HALO and meK‐means derive their priors from mathematical and physical laws—temporal ordering and biophysical constraints, respectively—while TkNA derives its priors from statistical consistency across multiple independent cohorts. Together, these emerging frameworks complement classical MR and prior‑knowledge networks, forming a diversified causal inference toolkit. Their application in livestock is still at an early stage, limited by the need for time‑series data, high‑quality single‑cell atlases, or multiple independent cohorts. Nevertheless, they represent promising directions for moving livestock multi‑omics from association‑centric descriptions toward genuine causal understanding.

To overcome the practical pitfalls of homology mapping, we propose several concrete strategies. First, for ambiguous one‑to‑many orthology relationships (e.g., one human gene mapping to two or more cow homologs), we recommend resolving mapping ambiguity using phylogenetic analysis and synteny conservation; only orthologs with conserved flanking gene order should be retained. Alternatively, the “high‑confidence” orthology set from Ensembl‑Compara, which filters one‑to‑many mappings based on reciprocal best hits and synonymy scores, can reduce systematic bias. Second, many livestock studies use non‑standard genome assemblies or custom annotation pipelines without providing explicit metadata (e.g., assembly version, gene annotation GTF source). For such datasets, homology mapping should not be performed directly; instead, we recommend converting coordinates to a standard reference assembly using liftOver before mapping. Third, as a future direction, supervised transfer learning (e.g., fine‑tuning graph neural networks pre‑trained on human protein‑protein interaction or gene regulatory network data with limited livestock multi‑omics data) offers a principled way to exploit cross‑species conservation while adapting to species‑specific connections. Finally, to build systematically curated livestock‑specific regulatory networks, we advocate community‑driven literature curation following the OmniPath model, starting with already experimentally validated livestock transcription factor–target pairs and protein complexes. These strategies collectively provide a practical roadmap for improving homology‑based inference in livestock.

Despite these methods, the core bottleneck remains the scarcity of species‑specific prior regulatory knowledge for livestock—such as phosphorylation substrate databases or transcription factor–target gene databases [134]. Livestock research still heavily relies on homology mapping for exploratory analyses; conclusions derived from such analyses, while possessing causal potential, still require experimental validation before practical application. To overcome this problem, methodological advances must be accompanied by the development of large‑scale systematic perturbation databases. Table 4 summarizes the three‑tier methodological spectrum, including the causal inference tier.

**TABLE 4:** Methodological Spectrum of Multi‐Omics Integration Strategies.

Integration Level	Method	Core Task & Key Features	Application / Potential in Livestock	References
Statistical correlation	Cross‐omics correlation: CCA, sCCA, rCCA, PLS, sPLS‐DA	Classical linear framework for omics covariation; interpretable but constrained by linearity and matched‐sample requirements.	rCCA for pig muscle transcriptome–fatty acid; DIABLO (sGCCA) for beef cattle multi‐tissue; sPLS‐DA for lamb methylome–transcriptome and pig breed metabolomics; Nellore carcass traits.	[143, 144, 145, 146, 147, 148, 149]
	Factor decomposition: NMF, MOFA+, iCluster, JIVE	Extracts latent factors from multi‐omics data, accommodating missing values and count data with Bayesian uncertainty quantification, though computationally demanding.	MOFA+ for linoleic acid metabolism and heat tolerance across cattle breeds; iCluster for methylation–transcriptome integration.	[92, 94, 95, 96, 97, 98, 150, 151]
	Network & kernel fusion: SNF, MKL, NEMO	Integrates sample similarity networks via SNF (missing‐data robust) and MKL (captures non‐linearity), though kernel choice critically affects performance.	FSBLUP for pig genomic prediction; MKL for beef and dairy cattle; SNF for pregnancy status and fertility genes in cattle.	[101, 104, 105, 152]
Machine learning	Supervised prediction: LASSO, RF, XGBoost, SVM	Excels at high‐dimensional feature selection and prediction with strong overfitting resistance (RF, XGBoost), but remains a non‐causal black box.	RF/XGBoost for feed efficiency in Nellore; poultry egg classification; LPA biomarker for PRRSV; multi‐tissue pig GRN.	[153, 154, 155]
	Unsupervised clustering & dim. reduction: PCA, t‐SNE, UMAP, K‐means, Hierarchical, DBSCAN	Uncovers hidden population structure without labels, but sensitive to hyperparameters and requires subjective cluster number choices.	PCA/UMAP for uterine metabolome–microbiome; t‐SNE for pig single‐cell atlas; K‐means/hierarchical for buffalo milk production; feed efficiency modules in cattle.	[156, 157, 158]
	Deep generative & graph/sequence models: VAEs, GANs, GNNs, Transformers	Enables joint latent representation, imputation, and batch correction with strong generalization from pre‐training, but computationally heavy and still lacks livestock‐specific models.	MLP‐VAE for Holstein genotype‐to‐phenotype; scVAEIT/MIDAS for mosaic single‐cell integration; FactVAE for multi‐omics clustering; CNEReg for ruminant GRN; scGPT/scFoundation potential for livestock.	[159, 160, 161, 162]
Causal inference	Mendelian Randomization (MR)	MR infers causal effects using genetic variants as instrumental variables, calibrated by population‐specific GWAS and QTL statistics. These priors are non‐transferable across species. Pleiotropy‐robust estimators address horizontal violations but not the prior's intrinsic population specificity.	Causal inference of host–microbiome interactions in dairy cattle for milk production traits and health traits	[141, 142]
	Prior‑knowledge network:CARNIVAL, CORNETO	CORNETO and CARNIVAL infer upstream causal regulators by integrating physical molecular interactions (PPI, TF–target, kinase–substrate) with transcriptomic footprints. Their priors derive from biophysical binding laws universal across organisms, making them cross‐species transferable, unlike MR's population‐specific statistical priors. Both face NP‐hard optimization.	(Potential) Framework directly applicable to livestock once species‑specific prior knowledge (TF‑target, enhancer) becomes available; CORNETO allows joint network inference across multiple samples/breeds	[163, 164]
	Enhancer‐driven GRN inference: SCENIC+	SCENIC+ reconstructs TF→enhancer→target gene cascades at single‐cell resolution by integrating chromatin accessibility with gene expression. TF binding motifs provide cross‐species transferable priors, while chromatin co‐accessibility patterns require species‐specific measurement. Requires paired scATAC‐seq and scRNA‐seq data.	(Potential) SCENIC+ for pig, cattle, and chicken single‐cell multi‐omics data; enhancer–gene validation currently relies on cross‐species homology mapping.	[165]
	Dynamics‐based causal inference: HALO, meK‐means	identifies distal regulatory interactions in time‐series single‐cell data using Granger causality, while meK‐means defines cell states through chemical master equations constrained by splicing kinetics. Both derive their priors from mathematical and physical laws universal across organisms.	(Potential) HALO for time‑series single‑cell data from livestock development; meK‑means for biophysical cell state definition.	[138, 141]
	Multi‐cohort causal network: TkNA	TkNA identifies causal master regulators by filtering spurious associations through statistical consistency across multiple independent cohorts, using Bayesian inference and network topology without requiring a pre‐compiled species‐specific interaction database.	(Potential) TkNA for multi‑cohort meta‑analysis of host‑microbiome interactions	[142]
	Metabolic fluxomics / Metabolic Control Analysis (MCA)	Measures metabolic network flux via stable isotope tracers, with causal evidence grounded in mass conservation and stoichiometric constraints—physical laws independent of statistical priors. MCA control coefficients quantify flux response to enzyme perturbation.	(Potential) predicts flux changes after genetic/nutritional interventions (e.g., milk fat, muscle amino acids); complements MR and network inference	[63, 64, 67]

## 7 Multimodal Sequencing

Although the integration strategies described above help us better leverage existing data to gain biological insights, fundamentally addressing the problem of data heterogeneity requires advances in sequencing technology itself. Multimodal omics sequencing tackles this challenge by simultaneously acquiring information from at least two omics dimensions from the same single cell or tissue sample, thereby eliminating sample heterogeneity and batch effects across omics layers. Current development and application of multimodal sequencing are primarily concentrated in three directions: single‐cell multimodal sequencing, spatial multimodal sequencing, and temporally dynamic multi‐omics sequencing.

Single‐cell multimodal sequencing is currently the most mature and widely applied direction. It overcomes the limitation of traditional single‐cell sequencing, which can only capture a single molecular layer, enabling the simultaneous dissection of multi‐dimensional omics information within the same cell. The evolution of spatial transcriptomics from spot‐level resolution to cellular or even subcellular resolution exemplifies this developmental trajectory. For instance, 10× Genomics' XENIUM technology has achieved in situ spatial transcriptomic sequencing at subcellular resolution [163]. Representative three‐omics methods include: TEA‐seq, which simultaneously analyzes chromatin accessibility, surface protein immunophenotype, and the transcriptome [164]; and NEAT‐seq, which simultaneously detects intranuclear protein abundance, chromatin accessibility, and gene expression [165].

On this basis, the 2026 CHARM technology achieved a milestone breakthrough [166]. This method captures, for the first time in the same cell, four key omics layers: 3D genome architecture, chromatin accessibility, histone modifications, and the transcriptome [CHARM]. CHARM builds on the authors’ previously developed HiRES (simultaneous Hi‑C and RNA sequencing) technology, further integrating a Tn5 transposase‑mediated chromatin tagging step to simultaneously target accessible chromatin regions and specific histone modifications. Using CHARM, the research team constructed a single‑cell 3D genome architecture map of 720 mouse embryonic stem cells at 5‑kb resolution. They found that chromatin accessibility regions form spatial clusters in the nucleus, enriched for super‑enhancers and cell‑type‑specific genes, and that these 3D clusters significantly enhance the coordination of gene expression within the clusters.

For temporally dynamic multi‐omics, a precedent for this approach can be seen in a recent study that analyzed transcriptome and non‑coding RNA profiles (mRNA, miRNA, lncRNA, circRNA) in the pectoral muscle of two chicken breeds across three late embryonic stages (embryonic days 17, 19, and 21), constructing a comprehensive ceRNA regulatory network that revealed breed‑specific energy metabolism pathways during hatching [167].

Overall, multimodal sequencing and temporal multi‐omics technologies address the problem of data heterogeneity in multi‐omics integration at its source. The paired data they generate can serve as high‐quality training inputs for machine learning and deep learning models, and can be directly utilized for the third‐tier causal inference. Although the application costs of these technologies in livestock remain relatively high, it is anticipated that prices will decrease and commercial applications will expand in the future, making this a direction worthy of attention. Table 5 presents representative multimodal sequencing technologies developed in recent years.

**TABLE 5:** Representative multi‐omics technologies for simultaneous profiling of multiple molecular omics layers.

Technology Platform	Omics Dimensions Simultaneously Detected	Resolution	Core Advantages	References
G&T‐seq	Genome + Transcriptome	Single‐cell	Simultaneous DNA and RNA profiling from the same cell, links genetic variation with expression	[168]
scM&T‐seq	DNA methylation + Transcriptome	Single‐cell	Parallel methylome and transcriptome profiling, reveals epigenetic‐expression coupling	[169]
REAP‐seq	Transcriptome + Protein	Single‐cell	Similar to CITE‐seq, scalable protein panel	[170]
TEA‐seq	Chromatin accessibility + Protein + Transcriptome	Single‐cell	Three modalities simultaneously, links epigenome, surface markers, and transcriptome	[171]
CHARM	3D genome conformation + Chromatin accessibility + Histone modification + Transcriptome	Single‑cell	Four omics dimensions simultaneously; captures 3D genome architecture, accessible chromatin, histone marks and gene expression in the same cell; enables integrative dissection of the three‑dimensional epigenome at single‑cell resolution	[172]
ASAP‐seq	Chromatin accessibility + Protein + Transcriptome + mtDNA	Single‐cell	Integrates epigenome, proteome, transcriptome, and mitochondrial variants	[173]
NEAT‐seq	Intracellular protein + Chromatin accessibility + Transcriptome	Single‐cell	Links signaling protein activity, chromatin state, and gene expression	[165]
DBiT‐seq	Transcriptome + Protein + Spatial location	10–50 µm	Spatial co‐detection of mRNA and protein, tissue section compatible	[174]
Spatial CITE‐seq	Transcriptome + Protein + Spatial location	10–50 µm	Spatial multi‐omics with antibody and transcript detection	[175]
Spatial ATAC‐RNA‐seq	Chromatin accessibility + Transcriptome + Spatial location	10–50 µm	Spatial epigenome and transcriptome from same tissue section	[176]
Spatial CUT&Tag‐RNA‐seq	Histone modification + Transcriptome + Spatial location	10–50 µm	Spatial profiling of histone modifications and gene expression	[177]
10× Genomics XENIUM	Transcriptome + Spatial location	Subcellular	In situ subcellular spatial transcriptomics, commercial platform	[178]
Spatial Molecular Imager	RNA (6,000 genes) + Protein (100+ proteins) + Spatial location	Subcellular	Ultra‐high multiplexing, subcellular resolution, FFPE compatible	[179]
scRepli‐RamDA‐seq (scRR‐seq)	DNA replication status + Full‐length total RNA (including non‐polyA RNA)	Single‐cell	Simultaneous profiling of DNA replication timing and transcriptome from same cell; enables precise S‐phase staging based on percent replicated genome; identifies S‐phase progression markers; haplotype‐specific analysis of replication and expression	[180]
TEMPOmap	Transcriptome kinetics (RNA synthesis & decay) + Subcellular RNA localization + Spatial location	Subcellular	Spatiotemporally resolved RNA dynamics at subcellular resolution; quantifies RNA synthesis, degradation and subcellular distribution simultaneously; enables kinetic profiling of transcriptome in intact tissues	[181]
scEdU‐seq	DNA replication speed + Genome‐wide replication timing + Transcriptome	Single‐cell	First method to quantify DNA replication fork speed at single‐cell level; links replication dynamics with gene expression; compatible with FFPE samples	[182]
SPACE‐seq	Chromatin accessibility + Transcriptome + Mitochondrial DNA mutations + Cell lineages	10–50 µm (Visium platform)	Integrates spatial epigenome, transcriptome, and clonal lineages; uses commercial Visium CytAssist platform for high‐throughput; enables lineage tracing in complex tissues	[183]
spatial‐DMT	DNA methylome + Transcriptome + Spatial location	Near single‐cell	First spatial technology for joint profiling of DNA methylation and transcriptome on same tissue section; uncovers spatiotemporal epigenetic regulation of gene expression	[184]
spatial‐Mux‐seq	2 Histone modifications + Chromatin accessibility + Transcriptome + Protein + Spatial location	Single‐cell	Five modalities simultaneously; maps histone marks (H3K27ac/H3K4me3), open chromatin, RNA, and proteins in situ; reveals epigenetic‐expression‐protein spatial coupling	[185]
DBiTplus	Transcriptome + Protein + Spatial location + Imaging‐based cell typing	10–50 µm	Integrates imaging‐based protein profiling (mxIF) with DBiT spatial transcriptomics; creates truly single‐cell‐level spatially resolved transcriptome atlases; guides transcriptome splitting by protein expression	[186]
TIP‐seq	Transcriptome + Intracellular Protein	Single‐cell	Simultaneous profiling of mRNA and intracellular proteins without prior cell manipulation or fixation; captures signaling protein activity and gene expression in native state; high sensitivity for low‐abundance proteins	[187]
nanoSPINS	Transcriptome + Proteome	Single‐cell	Droplet‐based high‐throughput method using centrifugation‐based mRNA transfer; combines RNA sequencing with isobaric labeling LC‐MS proteomics; generates global proteomic and transcriptomic profiles from same cell	[188]
Seq‐Scope‐X [109]	Transcriptome + Subcellular localization (nuclear vs cytoplasmic) + Spatial location	Subcellular (100–200 nm)	Enhanced expansion microscopy‐enabled spatial transcriptomics; resolves nuclear vs cytoplasmic mRNA distribution at subcellular level; pushes spatial resolution beyond diffusion limits	[189]

## 8 From Computational Inference to Experimental Validation: A Pragmatic Roadmap and Benchmarking Framework for Causal Inference in Livestock Multi‐Omics

The value of computational causal inference must ultimately be tested through experimental validation. However, livestock research is constrained by high costs, ethical considerations, and long generation intervals, making it difficult to directly replicate the high‑throughput perturbation screening models established in humans and mice. To address this practical challenge, we propose a phased, feasible roadmap integrated with established benchmarking frameworks to provide methodological support for causal inference in livestock.

### 8.1 Short Term: Cell‑Line Perturbation Validation

The most realistic starting point is CRISPRi perturbation in livestock cell lines. Whole‑genome CRISPR libraries have become commercially available (e.g., products from Cellecta) and openly accessible to the academic community in species such as pigs and chickens, enabling a single laboratory to validate tens to hundreds of candidate genes within months. Two complementary strategies can be pursued. The first is phenotype‑oriented pooled CRISPR screening, which measures sgRNA abundance changes under selective pressure (e.g., viral infection, drug treatment). This approach has been successfully applied in livestock to identify host factors essential for viral replication [69, 70]. The second is transcriptome‑oriented arrayed perturbation coupled with bulk RNA‑seq, exemplified by the Worm Perturb‑Seq method [190]. This method systematically perturbs 103 nuclear hormone receptors in C. elegans and uses the EmpirDE algorithm to correct false discovery rates via gene‑specific empirical null distributions. Its key advantages are: (i) bulk RNA‑seq costs are far lower than single‑cell approaches, making systematic validation of dozens of candidate genes affordable for individual laboratories; and (ii) the arrayed design allows precise control over experimental conditions, enabling the measurement of whole‑transcriptome responses rather than just cell survival. This paradigm is fully transferable to livestock cell lines and represents a realistic validation path that bridges conventional CRISPR screens and high‑resolution Perturb‑seq [191].

### 8.2 Medium Term: Organoid and Ex Vivo Model Validation

Livestock organoid technologies (intestinal, mammary, ovarian, etc.) are developing rapidly, and experience with organoid culture has been accumulating in livestock research. Organoids can partially recapitulate tissue structure and cell‑cell interactions in vitro, making them particularly suitable for validating causal hypotheses related to differentiation and development. Ex vivo tissue cultures (e.g., embryonic limb buds, ovarian tissue culture) provide complementary avenues for perturbation experiments at specific developmental stages.

### 8.3 Long Term: In Vivo Perturbation Screening

As technologies mature and costs further decline, a gradual transition to in vivo perturbation screening can be pursued, while leveraging transfer learning from high‑quality human/mouse perturbation data. Notably, livestock already possess gene‑edited individuals (e.g., PRRSV‑resistant pigs, double‑muscled cattle), and such “natural perturbation” resources can serve as valuable material for causal validation. The implementation roadmap is summarized in Table 6

**TABLE 6:** Implementation Roadmap from Causal Molecular Networks to Breeding Decisions.

Phase	Core Tasks	Key Technologies/ Methods	Required Resources / Data	Milestones
Phase 1 Causal discovery	Infer causal variants, regulons, or metabolic control points from existing multi‐omics data	MR, SCENIC+, CORNETO, (MCA)	Public databases (FarmGTEx, FAANG, AnimalTFDB); GWAS summary stats; species‐specific eQTLs if available	Prioritized list of causal targets (variants/genes/regulons/enzymes)
Phase 2 Cell‐line validation	CRISPRi or CRISPRa perturbation in livestock cell lines with bulk RNA‐seq to validate candidates	Arrayed CRISPR perturbation + bulk RNA‐seq (Worm Perturb‐Seq); EmpirDE statistical correction	Whole‐genome CRISPR libraries (commercial e.g., Cellecta, or open‐access e.g., Scishare); cell culture facilities;	Experimentally validated causal targets (drastically reduced false positives)
Phase 3 Organoid /ex vivo validation	Perturb targets in livestock organoids (intestinal, mammary, ovarian) or ex vivo tissue cultures	Organoid culture; lentiviral‐mediated gene editing; pharmacological inhibition	Organoid culture experience; access to ex vivo tissues (e.g., embryonic limb buds, ovaries)	Causal effects confirmed in more physiological contexts
Phase 4 Small‐scale in vivo validation	Gene editing or nutritional intervention in individual livestock (or model animals) to verify phenotypic effects	CRISPR/Cas9 gene editing (e.g., CD163 KO pigs); targeted nutritional interventions (e.g., flux tracing)	Gene‐edited animal facilities; ethical approval; nutrition/metabolism chambers	In vivo proof‐of‐causal‐effect (e.g., disease resistance, increased milk yield)
Phase 5 Breeding program integration	Incorporate validated causal targets into GS models (as prior weights), or use as fixed selection markers/embryo screening targets	GS models (BLUP, Bayesian, machine learning); embryo genotyping; whole‐genome selection chips	Breeding data management systems; high‐throughput genotyping platforms; population size >1000	Improved GS accuracy; accelerated genetic gain; commercial gene‐edited lines (e.g., FDA‐approved)

## 9 Benchmarking Framework: A Systematic Overview of Existing Methodological Evaluation Metrics

Even in the absence of livestock‐specific causal validation examples, mature benchmarking methodologies from human/mouse studies can serve as valuable references. Below we systematically summarize several benchmark frameworks that offer directly transferable evaluation metrics for livestock research.

### 9.1 Network Inference Benchmarking (DREAM Challenges)

The DREAM challenges are the most influential community‐wide benchmarking platform for gene regulatory network inference [192]. The long‐established evaluation system centers on two core metrics: AUPR (area under the precision‐recall curve) and AUROC (area under the receiver operating characteristic curve). AUPR is particularly sensitive to false positives in sparse networks, making it well‐suited for the inherently sparse structure of most real‐world regulatory networks. In multiple DREAM challenges (e.g., DREAM3, DREAM5), AUPR has been adopted as the primary evaluation metric. This metric system is directly applicable to assessing the performance of causal or gene regulatory network inference in livestock multi‐omics.

### 9.2 Single‐Cell Multi‐Omics Integration Benchmarks (scIB / scMultiBench)

A series of recent benchmarking studies have formed a multi‐level evaluation matrix. The scIB framework [193] established the first systematic evaluation metrics for single‐cell data integration. It divides assessment into two complementary dimensions: biological conservation (NMI, ARI, ASW_label, isolated‐label F1 score) and batch correction (ASW_batch, graph connectivity, kBET, PCR). This framework provides a reference for evaluating cell‐type annotation and integration quality in livestock single‐cell multi‐omics. A follow‐up study systematically evaluated 14 modal prediction and 18 integration algorithms across 47 datasets. A multi‐task benchmarking study [194] further comprehensively assessed integration methods across tasks such as dimensionality reduction, batch correction, cell classification, imputation, feature selection, and spatial alignment. More recently, the SCMBench study [195] focused on domain‐specific models versus foundation models, evaluating 23 methods on integration accuracy, biomarker detection, trajectory inference, and quantitative batch effect correction, revealing that foundation models generally underperform state‐of‐the‐art domain‐specific models in this domain.

### 9.3 Causal Inference Benchmark (CausalBench)

CausalBench [196] is a revolutionary causal evaluation framework. It constructs a benchmark suite based on large‐scale real single‐cell perturbation data, comprising more than 200 000 intervention data points from two cell lines (RPE1 and K562). CausalBench provides biology‐driven metrics and distribution‐based intervention metrics, making the evaluation of causal inference methods closer to real biological scenarios. A striking and cautionary finding is that, on real perturbation data, methods that use intervention information do not significantly outperform those using only observational data—a conclusion starkly different from synthetic benchmarks, highlighting the irreplaceable value of real‐world benchmarking. If livestock research can accumulate perturbation data of comparable scale in the future, the CausalBench paradigm will be directly transferable.

### 9.4 Genomic Prediction Benchmarks (EasyGeSe & NCG)

The field of genomic prediction has long relied on Pearson correlation and MSE for evaluation. However, a study [178] critically pointed out that using R^2^ as the model selection criterion can lead to erroneous choices; it recommended using test MSE for model selection and R^2^ for model performance assessment. The EasyGeSe tool [171] provides standardized genomic prediction datasets from 10 species, including pigs, with unified input formats and evaluation procedures, enabling fair and reproducible comparisons of genomic prediction methods. In a complementary direction, large‐scale perturbation models, such as the Large Perturbation Model (LPM), have been developed to learn joint representations of perturbations, readouts, and contexts from heterogeneous pooled perturbation data, enabling in silico prediction of post‐perturbation transcriptomes and inference of gene‐gene interaction networks [172]. Although LPM was trained exclusively on human cell lines, its methodological framework, which learns joint representations across heterogeneous perturbations, offers a valuable blueprint for designing similar benchmarking efforts in livestock once species‐specific perturbation data become available. More importantly, the Normalized Cumulative Gain (NCG) has been proposed as an alternative metric that directly measures the phenotypic gain achieved from individuals selected by the model, with its R implementation publicly available on GitHub.

Although livestock research currently lacks large‐scale systematic causal validation resources, the economic feasibility of the WPS paradigm and the mature benchmarking frameworks described above provide practical transitional pathways to fill this gap. We call on the community to prioritize the establishment of livestock‐specific benchmark datasets and to initiate community‐scale competitions modeled after the DREAM challenges and CausalBench, thereby advancing livestock multi‐omics causal inference from “concept” toward “verifiable” scientific practice. The benchmarking metrics discussed above are summarized in Table 7.

**TABLE 7:** Recommended Benchmarking Metrics and Application Scenarios for Livestock Multi‐Omics Integration and Causal Inference.

Task Module	Standard Evaluation Metrics	Detailed Interpretation	Inherent Limitations
Single‐cell integration & batch correction	NMI, ARI	NMI evaluates clustering‐label consistency; ARI corrects for chance	NMI insensitive to cluster number; ARI sensitive to class distribution
	ASW_label, Isolated‐label F1	ASW_label measures cell‐type separation; Isolated‐label F1 assesses rare cell type preservation	ASW_label depends on dimensionality reduction quality; Isolated‐label F1 requires true labels
	ASW_batch, Graph connectivity, kBET, PCR	ASW_batch and kBET evaluate batch mixing; Graph connectivity assesses cell‐type connectivity; PCR quantifies batch contribution to PC variance	kBET and Graph connectivity sensitive to noise and sample size; PCR requires specifying PC number
Classification & feature selection	Precision, Recall, F1‐score, AUROC, AUPR	F1‐score balances precision and recall; AUROC evaluates ranking; AUPR preferred for imbalanced data	Sensitive to class imbalance; AUROC biased toward majority class; AUPR requires well‐defined positive class
Gene regulatory network inference	AUPR, AUROC, Precision@k	AUPR preferred for sparse networks; AUROC evaluates overall ranking; Precision@k assesses top‐k edges	AUPR requires gold standard; AUROC can be optimistic in sparse networks; Precision@k depends on threshold
Genomic prediction & breeding gain	test MSE, R^2^, Predictive correlation, NCG	MSE for model selection; R^2^ for variance explained; Predictive correlation for rank accuracy; NCG directly measures phenotypic gain from selection	MSE not biologically intuitive; R^2^ alone insufficient for model selection; NCG requires actual phenotype data
Cross‐population generalization	ΔR^2^, AUPR decay	ΔR^2^ measures predictive performance drop across populations; AUPR decay quantifies network inference transferability	Requires multi‐breed/multi‐environment data
Causal inference	Intervention‐aware metrics, F1‐score on semi‐simulated data	Intervention‐aware metrics evaluate causal predictions against real perturbation data; F1 on known causal edges assesses accuracy on semi‐simulated benchmarks	Requires large‐scale perturbation data or careful simulation design

## 10 Application‐Oriented AI Multi‐Omics

While multimodal sequencing continues to generate high‐quality paired omics data, the fusion of generative pre‐trained AI models represented by scGPT with large language models is transforming the analytical paradigm of livestock multi‐omics, showing potential closely aligned with practical breeding applications. scGPT has undergone general pre‐training on tens of millions of single‐cell data points and, through lightweight fine‐tuning, can be adapted to tasks such as cell type annotation, multi‐omics integration, and gene regulatory network inference [126]. However, livestock multi‐omics datasets are generally limited by small sample sizes, strong population stratification, and severe batch effects, which expose directly transferred medical foundation model architectures to risks of overfitting and poor generalization.

In recent years, deep generative AI tailored to livestock breeding scenarios has made preliminary progress. The GRAD (Genetically Regulated Additive and Dominance) model extracts genetically regulated components from omics data. On real‐world data from 1494 Landrace pigs (105 of which had whole‐blood transcriptome sequencing), it improved prediction accuracy for age at 100 kg and total number of piglets born by 7.4% and 14.9%, respectively [197]. The MARS (Multi‐omics Annotation and Residual Split) model proposed a residual splitting strategy to mitigate overfitting and underfitting in deep learning and was validated on multiple pig traits [198]. In dairy cattle, the integration of deep learning with genomic selection has shown promise for predicting disease resistance, heat tolerance, and methane emission reduction, as systematically reviewed in several papers [199]. At the phenotyping level, Debnath et al. developed an end‐to‐end deep learning pipeline that automatically predicts bone breaking strength from chicken tibia X‐ray images. The model achieved a Pearson correlation up to 0.74 between predicted and measured values, and the predicted phenotype had a heritability (h^2^ ≈ 0.16) with a genetic correlation to actual bone strength exceeding 0.9, providing a non‐destructive proxy for large‐scale breeding selection [200].

In terms of benchmarking, Liu et al. performed a systematic multi‐task benchmark of 40 single‐cell multi‐modal omics integration methods, covering dimensionality reduction, batch correction, and clustering [194]. The EasyGeSe tool provides a standardized benchmarking platform for genomic prediction methods, with datasets covering 10 species including pigs, enabling fair comparison of different model prediction performance [171].

At the same time, the performance of single‐cell foundation models in zero‐shot livestock scenarios has clear limitations. A benchmarking study revealed that simpler machine learning models can be more efficient and adaptable on specific datasets than foundation models, and zero‐shot scFM embeddings performed poorly in recovering the distributional complexity of unobserved cells, suffering from a fundamental “temporal compression” bottleneck [201]. Moreover, batch effects remain a fundamental obstacle for single‐cell foundation models to achieve universal embeddings, and livestock data originating from multiple breeds, multiple farms, and multiple sequencing batches make direct transfer extremely challenging. Although dairy cattle accumulate relatively abundant and phenotype‐focused data, the integration of multi‑omics, model interpretability, and accessibility under low‐resource settings remain constraints.

To address these challenges, parameter‐efficient fine‐tuning (PEFT, e.g., LoRA, Adapter) fixes most parameters of a pre‐trained model and fine‐tunes only a small number of additional parameters, thereby avoiding catastrophic forgetting and overfitting on small‐sample livestock datasets. The value of scPEFT has already been demonstrated in cross‐species cell annotation tasks [202]. For cross‐population prediction, federated genomic prediction methods have been designed to handle non‐homogeneous genetic parameters across populations using effect transfer and retraining strategies. CycleGAN‐based cross‐breed small‐sample image generation has been used to expand training sets by generating synthetic Duroc images, achieving a mean average precision of 85.52% under a 10‐shot condition in pig detection. For model validation, we recommend using k×n cross‐validation (training on n breeds separately and testing on other breeds), leave‐one‐farm validation (evaluating generalization to new environments), and perturbation robustness tests (simulating batch effects) to systematically assess cross‑breed generalization. Moreover, cross‐farm deployment generalization often deteriorates significantly, highlighting the need to account for environmental heterogeneity when applying transfer learning in livestock settings. For evaluation metrics, we introduce the Normalized Cumulative Gain (NCG) as an alternative to traditional Pearson correlation or MSE, directly measuring the phenotypic gain achieved from individuals selected by the model; an R implementation is publicly available on GitHub. We also encourage researchers to share code and model weights, adopting a tiered openness strategy (core inference code must be open, data access may have moderate controls) to balance academic reproducibility and commercial interests.

**FIGURE 4:**
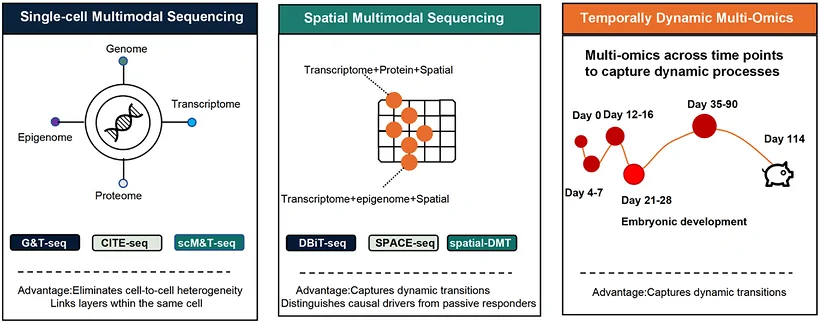
Future directions: multimodal sequencing and AI‐driven integrative breeding. Single‐cell multimodal sequencing measures multiple molecular layers within the same cell to eliminate cell population bias. Spatial multimodal sequencing retains in situ tissue positional information to distinguish causal drivers from secondary responses. Temporally dynamic multi‐omics tracks longitudinal molecular shifts across developmental or intervention timecourses, supporting temporal causal modeling of complex livestock traits.

Future efforts should focus on large‐scale pre‐training and architecture optimization using livestock‐specific omics data, and on building a standardized benchmarking system covering multiple species, tasks, and multi‐omics modalities. Cross‐species knowledge transfer and deep fusion of multimodal data represent potential breakthrough directions. Similar approaches, combined with the construction and sharing of large‐scale multi‐species reference atlases for livestock, could help remedy current shortcomings in data accumulation and model adaptability. Ultimately, establishing a benchmarking system dedicated to livestock breeding will ensure the reliability and reproducibility of model results and drive AI technology from the laboratory toward breeding applications. The integration of multimodal sequencing and AI foundation models toward precision breeding is conceptualized in Figure 4.

## 11 From Causal Molecular Networks to Breeding Decisions: Implementation Pathways and Industrial Translation

The pervasive difficulties in current livestock multi‐omics research have been detailed in Sections 4 and 5: a large number of studies compress high‐throughput data into simple lists of differentially expressed molecules, discarding rich information on regulatory dynamics, pathway crosstalk, and network interactions. Even the few studies that incorporate machine learning largely remain at the level of “association prediction,” still outputting correlational signals. This situation has led to a lack of clear data‐to‐decision translation paradigms in the field. The framework proposed in this review is designed precisely to address this problem—its ultimate output is a set of causally validated molecular targets or regulatory circuits, not yet another list of differentially expressed molecules.

The three tiers of our framework constitute a progressive analytical path from raw data to breeding decision inputs. The first tier (statistical association) identifies cross‐omics covariation patterns, answering whether variables are associated. The second tier (machine learning) handles data heterogeneity, performs dimensionality reduction and feature selection, and extracts testable candidate signals from noise. The third tier (causal inference) examines the causal direction of candidate signals, excludes confounders, and establishes intervention‐ready regulatory relationships. Only targets that pass causal scrutiny merit investment of breeding resources for subsequent validation and translation. Thus, the framework is not a simple stacking of methods but a hierarchical filtering system from data to decisions.

In breeding practice, the outputs of causal molecular networks can be directly applied along two parallel pathways. The selection pathway centers on genomic selection (GS), converting the prioritized gene sets or key QTL/eQTL information from causal networks into prior weights for GS models. Empirical evidence supports this pathway: Shi et al. [197] integrated PigGTEx QTLs and eQTLs as prior weights, significantly improving the prediction accuracy of GS models in 2295 Yorkshire pigs; Qian et al. [203] used the AbGP framework with a self‐attention mechanism to select key SNPs, compressing causal information to a practically usable scale (approximately 0.4% of SNPs) while maintaining or even improving prediction accuracy. The creation pathway points to gene editing and embryo engineering, using key regulatory nodes discovered by causal networks as primary CRISPR targets to reduce trial‐and‐error. A typical example is the CD163 gene: after being confirmed as a critical host factor for PRRSV infection, multiple teams worldwide used CRISPR/Cas9 to knock out CD163, generating PRRSV‑resistant gene‐edited pigs. In April 2025, the FDA approved the CD163ΔE7 intentional genomic alteration (IGA) in PRRSV‐resistant pigs for use in the U.S. food supply chain (21 CFR§528.2000; 90 FR 40970), marking the first commercialized gene‐edited livestock trait based on a validated causal target for a major agricultural disease. Although the technical systems of the two pathways differ, they both depend on reliably causal‐validated molecular targets—the core output of the third tier of our framework.

Currently, the direct industrial application of causal inference in livestock remains in its infancy. This is more a reflection of the field's developmental stage than a limitation of the framework itself. Nevertheless, positive signals are emerging: the FarmGTEx project is rapidly improving multi‐tissue eQTL atlases for cattle, pigs, chickens, and other species, providing critical prior knowledge for causal inference; resources such as FAANG and AnimalTFDB are filling functional annotation gaps; whole‐genome CRISPR libraries have become commercially available (e.g., from Cellecta); the cost of high‐throughput sequencing continues to decline, and adaptation to agricultural application scenarios is progressing. These developments indicate that the technical chain from causal inference to breeding application is moving from concept toward reality. The purpose of this section is to provide a systematic roadmap for this ongoing paradigm shift.

## 12 Conclusions and Perspectives

Livestock multi‑omics research has successfully progressed from genomics to multi‑dimensional, cross‑system analysis. However, to fully realize the potential of multi‑omics technologies in livestock genetics and breeding, systematic progress is still required in three core domains: underlying data infrastructure, methodological innovation, and industrial translation.

At the infrastructure level, livestock multi‑omics data still face challenges such as high sample heterogeneity, non‑standardized sequencing and analysis pipelines, and a severe lack of functional validation data. Building standardized databases and strengthening functional annotation are key priorities. We particularly recommend adopting the Worm Perturb‑Seq paradigm to develop arrayed CRISPR perturbation combined with bulk RNA‑seq validation workflows in livestock cell lines, thereby filling perturbation resource gaps in a cost‑effective and accessible manner. Concurrently, promoting cross‑team collaboration, developing standardized analysis workflows and data‑sharing platforms—together with systematic laboratory benchmarking and cross‑platform data calibration—will help mitigate the impact of data heterogeneity.

At the methodological level, a shift from correlation‑based analysis toward causal interpretation is urgently needed. The three‑tier framework proposed in this review (statistical association → machine learning → causal inference) provides an actionable pathway for this transition. We recommend prioritizing livestock‑specific benchmarking studies, adopting internationally recognized evaluation metrics such as NMI, ARI, and AUPR, and constructing “gold standard” test sets using semi‑simulated data. For scenarios such as local breeds with small sample sizes and scarce multi‑omics resources, cross‑sample joint inference frameworks like CORNETO are recommended as transitional solutions to move from “data waste” toward “data utilization.”

At the application level, translating multi‑omics research outcomes into breeding decisions requires not only data sharing and standardized workflows but also rigorous phenotyping. The prioritized targets output by causal networks can be implemented through two pathways: first, as prior weights for genomic selection models; second, as candidate targets for gene editing. With the continued improvement of infrastructures such as FarmGTEx and FAANG, and the ongoing decline in high‑throughput sequencing costs, the systematic integration of multi‑omics data will undoubtedly become a core tool for dissecting the genetic mechanisms of complex traits in livestock, for breeding new varieties with independent intellectual property rights, and for providing valuable resources for genetic research in non‑model organisms.

## Author Contributions

**Zhongyu Wang**: conceptualization, methodology, writing – review and editing, writing – original draft, validation, supervision, resources, software. **Fen Li**: writing – review and editing, supervision, resources. **Jiying Wen**: conceptualization, methodology, data curation, visualization, validation, writing – original draft, writing – review and editing, investigation. **Jieping Huang**: writing – review and editing, supervision, resources. **Yun Ma**: funding acquisition, writing – original draft, writing – review and editing, resources, supervision, data curation, conceptualization, project administration, formal analysis. **Ningbo Chen**: conceptualization, methodology, writing – review and editing, writing – original draft, resources, supervision, data curation, funding acquisition, project administration, formal analysis.

## Conflicts of Interest

The authors declare no conflict of interest.

## Data Availability

Data sharing not applicable to this article as no datasets were generated or analysed during the current study.
